# Unveiling Photo-Thermal-Electrical Performance in Robust, Self-Healing, and Anti-Freezing Cellulose-MXene Eutectogels for Advanced Hemostasis

**DOI:** 10.1007/s40820-026-02283-9

**Published:** 2026-07-20

**Authors:** Chuang Jiang, Hengli Ning, Wei Liu, Zhikun Li, Huiwu Zhu, Long Li, Qingxi Hou, Chaoji Chen, Bowen Cheng

**Affiliations:** 1 State Key Laboratory of Bio-Based Fiber Materials, China Textile Industry Key Laboratory of High-Performance Fibers Wet-Laid Nonwoven Materials, Tianjin Key Laboratory of Pulp & Paper, Tianjin University of Science and Technology, Tianjin, 300457 People’s Republic of China; 2 Hubei Biomass-Resource Chemistry and Environmental Biotechnology Key Laboratory, Hubei Provincial Engineering Research Center of Emerging Functional Coating Materials, School of Resource and Environmental Sciences, Wuhan University, Wuhan, 430079 People’s Republic of China

**Keywords:** Cellulose, Deep eutectic solvents, MXene, Photothermoelectric eutectogel, Hemostasis

## Abstract

Uncontrolled hemorrhage, resulting from trauma or surgery, presents a critical challenge in medical care. This study introduces a novel eutectogel, a multifunctional material synthesized using choline chloride/phytic acid derived deep eutectic solvents (CP-DES)-mediated cellulose-MXene polyacrylamide, aimed at addressing hemostatic needs. The eutectogel combines photothermal and thermoelectric effects to accelerate hemorrhage control, significantly reducing blood loss and hemostasis time. Unlike existing hemostatic materials, our design leverages the synergistic effects of photothermal heating and thermoelectric current, enhancing coagulation and promoting tissue repair. The innovation lies in the integration of DES to stabilize MXene, optimizing its photothermal and thermoelectric properties, and enabling rapid gelation, self-healing, and anti-freezing capabilities. In vitro and in vivo tests demonstrate the material’s superior performance in hemostasis compared to traditional gauze, highlighting its potential for trauma care and surgical applications. This approach sets a new paradigm in the development of advanced, multifunctional biomedical materials for hemostasis and beyond.

**Supplementary Information:**

The online version contains supplementary material available at 10.1007/s40820-026-02283-9.

## Highlights

- A deep eutectic solvent-mediated strategy integrates cellulose and MXene into a polyacrylamide network, enabling rapid gelation, and long-term MXene stabilization (>690 days).
- The eutectogel exhibits tissue-adhesive, self-healing, anti-freezing properties, high ionic conductivity, and efficient photothermal conversion, enabling multimodal sensing and synergistic photothermoelectric hemostasis.
- First demonstration of synergistic photothermoelectric hemostasis, where localized photothermal heating and Seebeck-driven microcurrents synergistically accelerate coagulation, achieving superior hemostatic performance with significantly reduced blood loss and hemostasis time.

**Supplementary Information:**

The online version contains supplementary material available at 10.1007/s40820-026-02283-9.

## Graphical abstract

**Figure:**
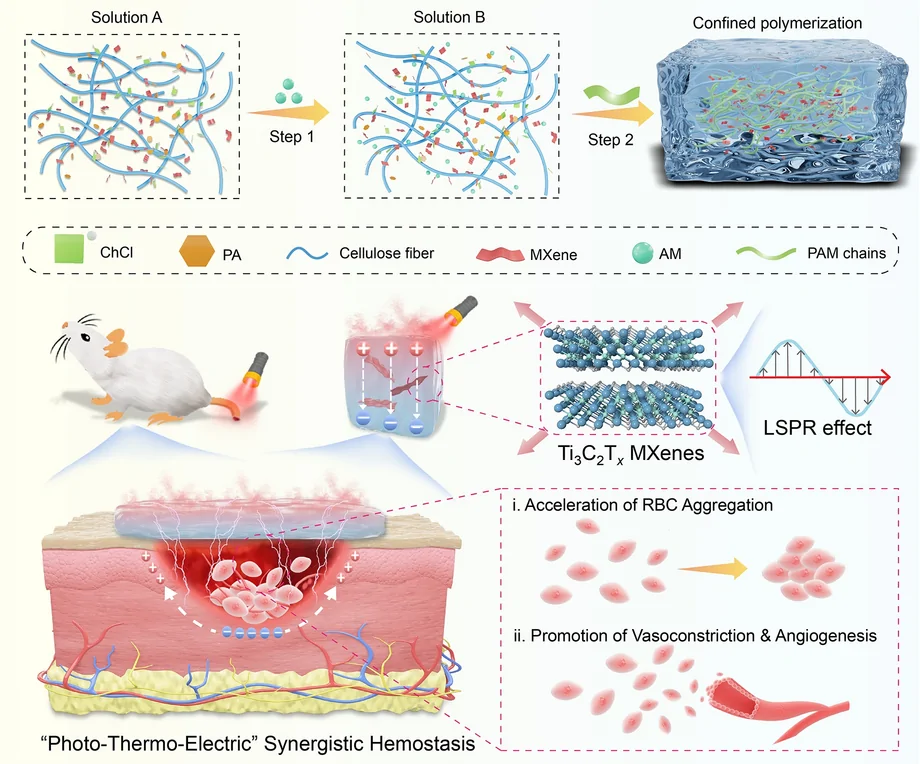

## Introduction

Uncontrolled hemorrhage, arising from traumatic injuries or surgical procedures, presents a critical medical challenge and imposes a substantial burden on global healthcare systems [1]. Effective and prompt management of life-threatening hemorrhage is essential for gaining critical time for subsequent medical interventions and significantly reducing mortality rates [2]. To date, a variety of hemostatic materials including gels [3], gauzes [4], sponges [5], and bandages [6], have been developed from both synthetic and natural origins. Eutectogels incorporate deep eutectic solvents (DES) as effective liquid active components and inherits their excellent biocompatibility, adhesion, intrinsic conductivity, and environmental stability, making it a potential hemostatic material [7]. Compared with traditional hydrogels, eutectogels offer distinct advantages, including excellent anti‑freezing capability, resistance to dehydration, intrinsic ionic conductivity, and often self‑healing behavior, owing to the unique properties of deep eutectic solvents (DES). However, the delicate design of innovative eutectogels to unlock their full hemostatic potential remains a major challenge.

Recent advances at the intersection of materials science and biomedical engineering have spurred the development of active hemostatic technologies, such as photothermal, thermoelectric, and micro-pulse systems [8, 9]. These technologies offer controllable energy delivery, high spatial precision, and remarkable sensitivity, providing new insights for the design of eutectogels [10]. For instance, Liu et al. [11]. developed a eutectogel microneedle based on MXene with photothermal response and antioxidant activity, which promotes wound closure and improves angiogenesis, providing a solution for precise wound management. Yu et al. [12]. reported a dual-network conductive eutectogel that, under exogenous electrical stimulation, reduces inflammation, promotes cell proliferation and migration, and enhances collagen deposition, angiogenesis, and skin tissue remodeling, demonstrating its potential as a dressing for severe burn wound treatment. Despite these individual advances, the potential synergy between photothermal and thermoelectric modalities remains unexplored. Thus, incorporating photothermoelectric agents into the design of eutectogels merges photothermal and thermoelectric approaches, which reduces energy loss and combines the benefits of thermal and electrical stimulation to improve hemostatic performance.

The rational selection of photothermoelectric agents and their synergistic interaction with the eutectogel components are crucial for fully harnessing their hemostatic potential. Ideal photothermoelectric agents should exhibit rapid photo-responsiveness, high biosafety, and long-term stability under ambient storage. To this end, a variety of materials, such as carbon-based nanomaterials [13], black TiO_2–*x*_ [14], and topological insulators [15], have been actively investigated. Among them, MXenes, a family of graphene-like two-dimensional compounds, have attracted growing interest due to their pronounced local surface plasmon resonance (LSPR), which stems from their semi-metallic electronic structure and ultrahigh electrical conductivity. The unique combination of mechanical robustness, high aspect ratio, and tunable surface chemistry positions MXenes as a premier material for diverse applications, such as catalysis [16], biomedicine [17], flexible electronics [18], and energy storage [19]. Notably, MXenes-based devices are vulnerable to oxidation in ambient conditions (e.g., air, moisture, or light), undermining their structure and LSPR performance. To address this, integration into DES-based eutectogels presents a sustainable and scalable strategy that effectively shields MXenes from degradation, thereby retaining their key photothermal and thermoelectric properties [20].

**Fig. 1:**
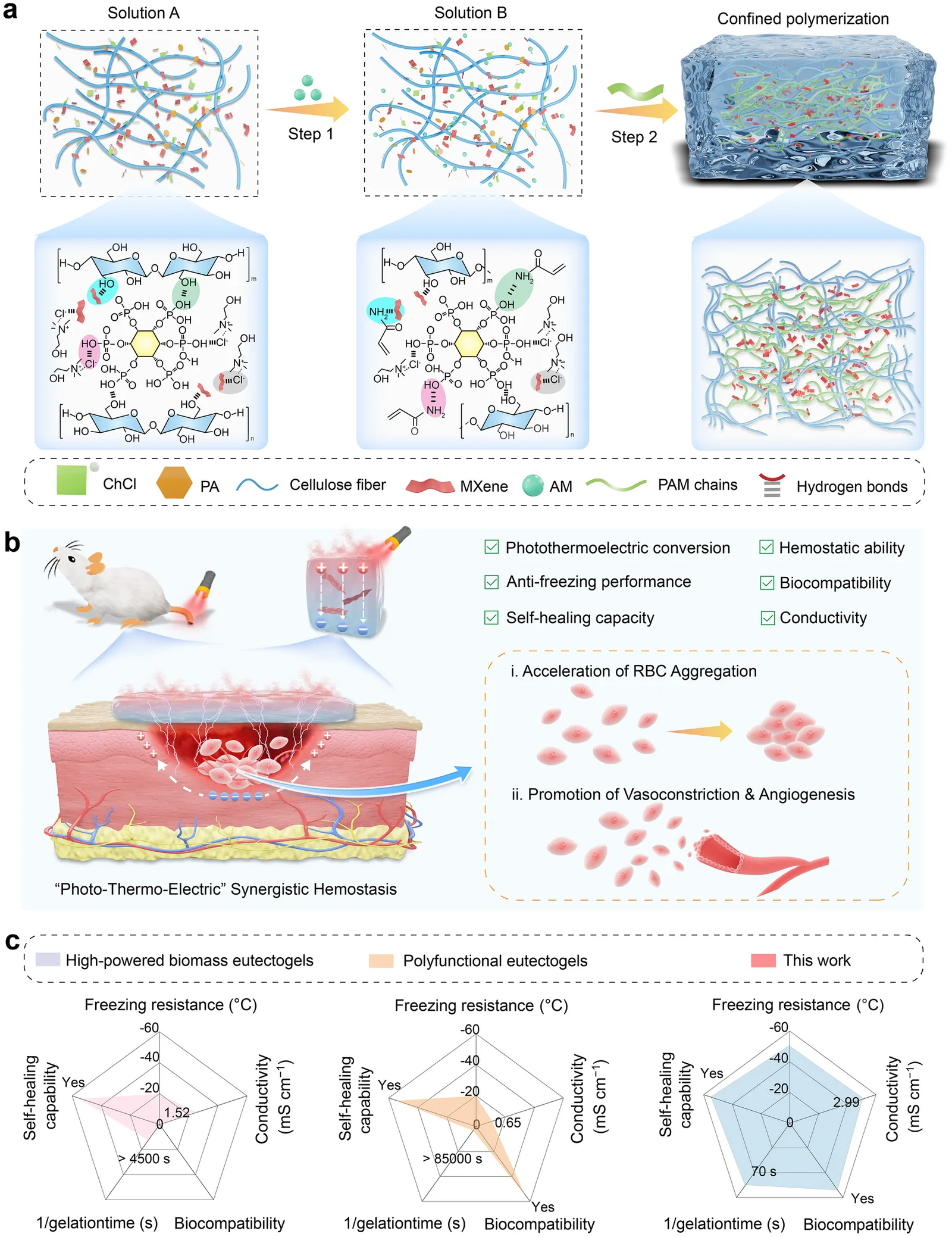
Fabrication strategy, synergistic hemostatic mechanism, and comprehensive performance advantages of DCMP eutectogels. **a** Schematic procedure for fabricating DCMP eutectogels. **b** Chematic illustration of synergistic photothermoelectric hemostasis mechanism of DCMP eutectogels: (i) Acceleration of red blood cell aggregation, and (ii) promotion of vasoconstriction and angiogenesis under photothermal and thermoelectric stimulation. **c** A comparison of key properties: gelation time, freezing resistance, self-healing capability, conductivity, and biocompatibility, among high-powered biomass eutectogels, polyfunctional eutectogels, and the DCMP eutectogels

Herein, we present a multifunctional DES-mediated cellulose-MXene polyacrylamide (DCMP) eutectogel, fabricated via a strategy that integrates cellulose and MXene into a polyacrylamide network within a choline chloride/phytic acid-based DES (CP-DES) (Fig. 1a). This DES serves a quadruple role as a green solvent, a dynamic hydrogen bonding matrix, an antioxidative microenvironment, and a cryoprotectant. This present design overcomes the traditional trade-off between mechanical strength and functional utility, yielding a material with rapid gelation within 70 s (Fig. S1a), a stable precursor solution for long-term storage and large-scale processing (Fig. S1b), high electrical conductivity, exceptional toughness, autonomous self-healing, and frost resistance at − 50 °C (Fig. 1b). To the best of our knowledge, no previous study has reported a hemostatic strategy that integrates photothermal and thermoelectric generation within a single material platform, making this work the first demonstration of a synergistic multimodal photothermoelectric approach to hemorrhage control. This unique attribute, together with its superior performance over existing eutectogels (Fig. 1c), establishes a new paradigm for versatile biomedical applications, particularly in photothermoelectric hemostasis.

## Experimental Section

### Preparation of DCMP Eutectogel

The mixture of phytic acid (PA) and choline chloride (ChCl) at a mass ratio of 1:1 was placed in a beaker, and heated in a 90 °C water bath for 1 h with stirring to obtain the DES. The as-prepared cellulose fibers were added to the prepared DES, in which the mass ratio of DES to the cellulose fiber was kept at 20:1. Subsequently, 40 mg of MXene powder was introduced, and the mixture was ultrasonicated to obtain a uniform dispersion. Separately, an aqueous solution containing acrylamide(AM) (6 g in 10 mL H_2_O), N,N'-Methylenebisacrylamide (MBA), and Ammonium persulfate (APS) was prepared. This solution was mixed into the MXene dispersion, and the final mixture was rapidly transferred to molds for gelation, producing the DCMP eutectogel.

### Characterizations of the DCMP Eutectogel

The chemical structures, micro-morphologies, thermal stability, mechanical robustness, and optical properties of the synthesized eutectogels were systematically investigated. Specifically, the molecular interactions, compositional distribution, and water states were elucidated using Fourier transform infrared spectroscopy (FTIR), variable temperature FTIR (VT-FTIR), Raman mapping, low-field nuclear magnetic resonance (LF-NMR), and X-ray diffraction (XRD). The surface and cross sectional microstructures were observed via SEM. Thermal and mechanical behaviors were evaluated through thermogravimetric analysis (TGA), differential scanning calorimetry (DSC), and comprehensive tensile/compression testing. Furthermore, the optoelectronic, photothermal conversion, and versatile strain-sensing performances of the eutectogels for human motion monitoring were systematically recorded. Detailed descriptions of the characterization instruments, specific experimental parameters, and testing protocols are provided in the Supporting Information.

### Swelling Behavior Tests

The original eutectogel sample was immersed in deionized water. After sufficient swelling, the eutectogel was removed, and any excess surface solution was gently blotted away using filter paper. The mass of the eutectogel was measured both before and after swelling. The swelling ratio (SR) is then calculated using the Eq. (1):

1 $$SR = ({w}_{i}-{w}_{0})/{w}_{0}\times 100\%$$

where *w*_*i*_ and *w*_0_ represent the weight of test sample at time = *i* and 0, respectively.

### Calculation of Photothermal Conversion Efficiency

To evaluate the light-to-heat conversion performance, the photothermal conversion efficiency (PCE) of test sample was measured under 0.1 W cm^–2^ incident light. The calculation followed the established method described by Liang et al. [21]. Detailed steps are shown in Supporting Information.

### Cytotoxicity Test

The in vitro cytotoxicity and biocompatibility of the eutectogels were evaluated using L929 fibroblasts. The cell viability and proliferation after incubation with various concentrations of gel extracts were quantitatively assessed via the Cell Counting Kit-8 (CCK-8) assay. To further visualize the cytocompatibility, live/dead fluorescence staining (Calcein AM/PI) was performed and observed using confocal laser scanning microscopy (CLSM). Detailed experimental protocols are provided in the Supporting Information.

### Hemolysis Test

The hemocompatibility of the samples was evaluated via a standard in vitro hemolysis assay using mouse erythrocytes. The structural integrity of the red blood cells upon contact with the materials was quantitatively assessed by measuring the absorbance of the released hemoglobin in the supernatant at 540 nm. Detailed procedures for the preparation of the erythrocyte suspension, assay protocols, and the calculation of the hemolysis rate are provided in the Supporting Information.

### In Vitro Coagulation Test of the DCMP Eutectogel

The in vitro hemostatic capability of the eutectogels was quantitatively evaluated using a dynamic whole blood clotting assay. The blood clotting index (BCI) was determined by monitoring the absorbance of free hemoglobin (at 540 nm) released from uncoagulated red blood cells after incubation with recalcified whole blood. Commercial medical gauze was utilized as the control. A lower BCI value indicates a superior blood clotting ability. Detailed experimental procedures, including physiological simulation parameters and the BCI calculation, are provided in the Supporting Information.

### In Vivo Hemostatic Assay of the DCMP Eutectogel

The in vivo hemostatic efficacy of the DCMP eutectogel was comprehensively evaluated utilizing two distinct bleeding paradigms: a rat tail amputation model and a severe rat liver perforation model. To highlight the photothermal-assisted rapid coagulation capability of the materials, the performance of the eutectogels under xenon lamp irradiation was systematically compared against untreated negative controls, standard commercial gauze, and xenon lamp irradiation alone. The clinical hemostatic potential was quantitatively assessed by continuously monitoring the total hemostasis time and overall bleeding volume. All animal procedures complied with established ethical standards. Detailed descriptions of the animal models, surgical protocols, and ethical approvals are provided in the Supporting Information.

### Statistical Analysis

All statistical analysis was executed utilizing Origin 2021 or GraphPad Prism 9.0 software. The data were presented as mean values ± standard deviation. Independent t test and one-way ANOVA followed by Tukey’s multiple comparison test were used to determine statistical significance between two or more groups, respectively. The group comparisons were conducted employing Student’s t-test, with the statistical significance was determined to be *p* < 0.05.

## Results and Discussion

### Preparation of DCMP Eutectogel

DCMP eutectogel was synthesized using a CP-DES that enabled the in situ dissociation of cellulose and subsequent confined polymerization of acrylamide (AM). Unlike common DES, this CP‑DES offers a unique combination of high conductivity, anti‑freezing properties, cellulose dissociation, and antioxidative protection for MXene [22]. Briefly, cellulose fibers and MXene were sequentially added to CP-DES, followed by sonication to obtain a homogeneous mixture. AM was then dissolved in distilled water and introduced to the mixture. This solution was quickly poured into molds, where spontaneous gelation occurred, forming eutectogels without requiring external energy input. In contrast to conventional polymerization that requires external activation such as high temperatures or ultraviolet irradiation, the eutectogels prepared in this study polymerized autonomously within a short period after the addition of AM, the crosslinking agent, and initiator. Within 70 s, the mixture formed a mechanically robust, stretchable eutectogel with no liquid residue, confirming successful gelation (Fig. S2). Compared with previously reported eutectogels, the DCMP eutectogel developed in this work stands out owing to its exceptional anti-freezing performance down to − 50 °C, high ionic conductivity of 2.99 mS cm^−1^, rapid gelation, excellent biocompatibility, and robust self-healing capability (Fig. 1c and Table S1).

A key innovation of this work is the design of a CP-DES that serves an inherently multifunctional role, which is directly capitalized upon in the eutectogel preparation. Firstly, CP-DES plays a dual role as both solvent and structural modifier that enables in situ cellulose dissociation, enhancing mechanical properties and lowering energy consumption. CP-DES effectively breaks the dense intermolecular and intramolecular hydrogen bonds in cellulose, causing it to dissociate in situ into flexible chains. These long, flexible cellulose chains become highly entangled with PAM chains, forming a robust, interpenetrating physical framework. Furthermore, the dissociation process exposes abundant hydroxyl groups on the cellulose backbone [23]. These hydroxyl groups act as multiple crosslinking sites, forming dense dynamic hydrogen bonds with PAM, MXene, and DES components, which enhances interfacial bonding and thereby improves the mechanical properties of the eutectogel [24]. In addition, the intercalation of cellulose with 2D MXene effectively prevents the restacking of MXene nanosheets [25]. To investigate the effect of CP-DES on cellulose, the CP-DES pretreated and untreated cellulose fibers were compared. As shown in SEM images, untreated cellulose fibers remain its long, robust form (Fig. S3a, b), whereas the cellulose pretreated with DES exhibit significantly shorter lengths and smaller diameters (Fig. S3c, d). The cellulose fibers pretreated with CP-DES exhibits primarily diameters of 10–20 µm, whereas the untreated ones range from 30–40 µm (Fig. S3e). This size reduction is consistent with SEM observations. This indicates that CP-DES pretreatment disintegrates cellulose into finer and shorter fibers. This morphology enhances its integration with MXene and weakens the interlayer forces of MXene, thus facilitating the subsequent polymerization. Secondly, strong hydrogen bonds form between the DES and MXene, enabling the DES to act as an intercalating agent that facilitates its delamination. This dynamic hydrogen bonding network ensures uniform MXene dispersion and spatially confines the polymerization of acrylamide, leading to a structurally homogeneous and mechanically robust eutectogel with enhanced photothermal conversion efficiency. Thirdly, the CP-DES creates an antioxidative microenvironment that prevents MXene oxidation through dual mechanisms of oxygen scavenging and physical barrier formation. This protection is critical for preserving the electrical and photothermal performance of MXene, thus maintaining its dispersion and chemical stability for over 690 days (Fig. S4).

### Characterization of DCMP Eutectogel

**Fig. 2:**
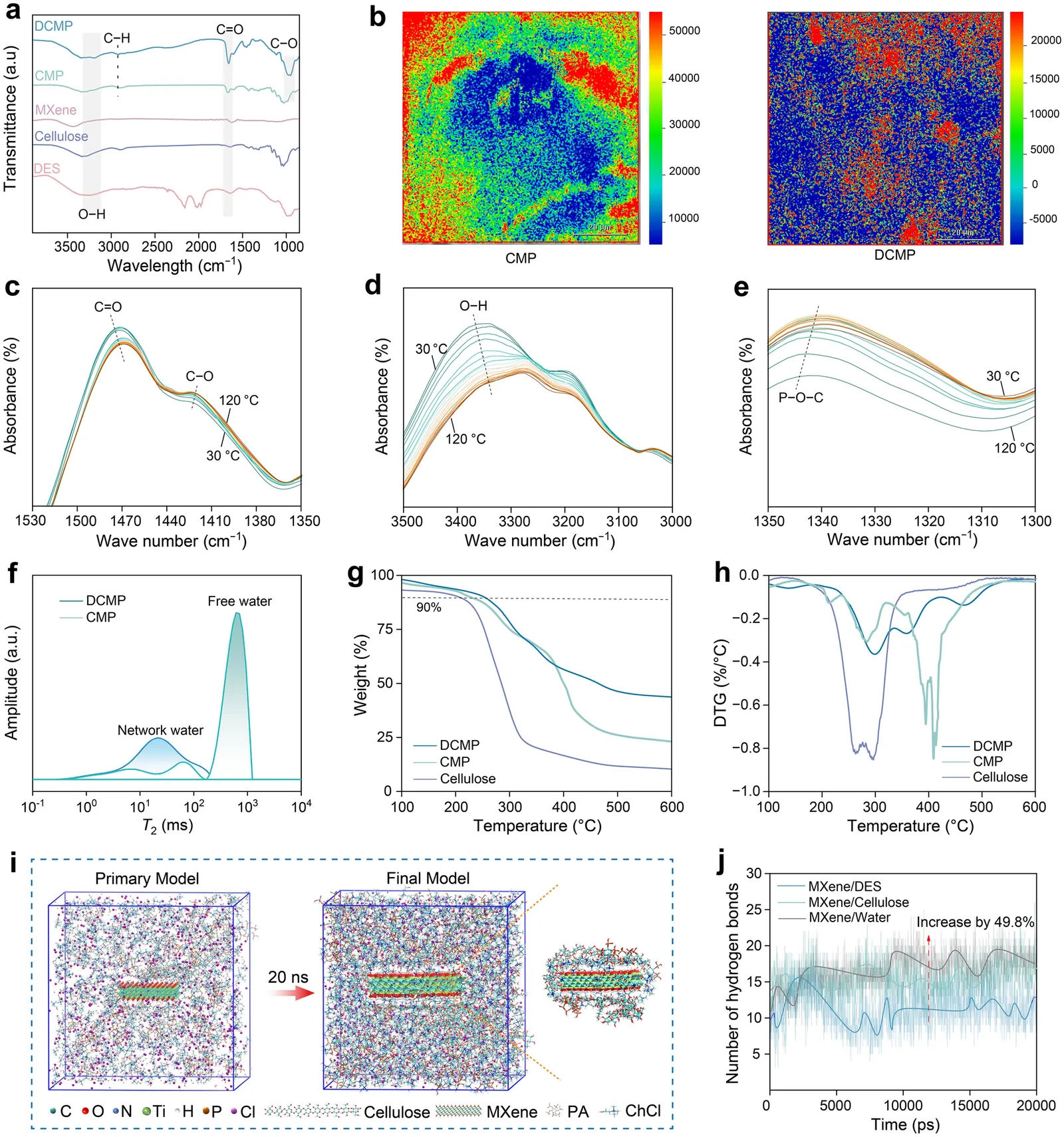
Structural characterization and intermolecular interaction dynamics of DCMP eutectogel. **a** FTIR spectra of samples. **b** 2D mapping of Raman of CMP and DCMP. **c-e** VT-FTIR spectra of DCMP recorded in the range of **c** 1350–1530 cm^–1^, **d** 3000–3500 cm^–1^, and **e** 1300–1350 cm^–1^. **f** LF-NMR curves of the samples. **g** TGA and** h** DTG curves of samples. **i** Initial and final state models of DCMP and the meaning of each molecular model. **j** Variation of the number of hydrogen bonds in the DCMP system with time

FTIR was used to investigate the structural changes of test samples, including DCMP, CMP (cellulose-MXene polyacrylamide), cellulose fibers, and MXene. The peaks at 3340, 2900, and 1370 cm^–1^ correspond to O–H stretching, C–H asymmetric stretching, and C–H bending vibrations of cellulose fibers, respectively (Fig. 2a) [26]. Compared to cellulose fibers, both CMP and DCMP display weakened peaks and a broadened O–H stretching band at 3340 cm^–1^, indicating esterification occurs on cellulose fibers [27]. The appearance of stronger peaks in DCMP at 1600–1000 cm^–^^1^ confirms the successful integration of MXene into the polymer network. Additionally, the observed shift of the O–H band from 1648 to 1624 cm^–^^1^ is attributed to interactions with the DES. This finding highlights the active role of the CP-DES in facilitating chemical modifications and enhancing the overall system interactions [28].

XRD analysis was performed to investigate the crystal structures of test samples. The XRD spectrum of the DCMP eutectogel shows the characteristic (002) peak of MXene at 2θ = 5.62°, a significant shift from 2θ = 7.10° in pure MXene (Fig. S5a). This shift corresponds to an increased interlayer spacing, attributed to the concurrent intercalation of DES and cellulose that weakens the interlayer forces and promotes exfoliation during sonication. These results confirm effective MXene delamination, and highlight the key role of DES in achieving a homogeneous composite.

Then, further insights into the component interactions were obtained through Raman spectroscopy (Fig. S5b). The Raman spectrum of MXene was characterized by a plasmon resonance peak at 150 cm^–^^1^, alongside in-plane (*E*_*g*_) and out-of-plane (*A*_*1g*_) vibrational modes in the ranges of 230–470 and 580–730 cm^–^^1^, respectively. In the DCMP spectrum, a strong peak at 168 cm^–1^ corresponds to the in-plane and out-of-plane vibrations of Ti and C atoms in MXene [29].

Raman mapping reveals a stark contrast in signal distribution between CMP and DCMP eutectogel (Fig. 2b). The CMP sample exhibits significant intensity variation, reflecting poor component homogeneity. In contrast, DCMP shows a highly uniform signal distribution. This is attributed to the CP-DES-mediated interactions, including hydrogen bonding with cellulose and electrostatic forces from choline chloride [30], which optimize the internal structure [31]. This structural homogeneity is crucial for the enhanced mechanical and functional properties of the eutectogel.

SEM was used to further observe the surface morphology of DCMP. DCMP exhibits a uniform large-pore structure (Fig. S6a), which plays a critical role in applications such as blood uptake and concentration [32]. This network structure is the result of interactions between MXene and cellulose, which also traps the CP-DES within the material. PA in the CP-DES, known for its conductive capacity, facilitates electron transport, which is essential for the photothermal and electrical functionalities of the resultant eutectogels. In addition, energy dispersive spectroscopy (EDS) elemental mapping reveals a uniform distribution of C, N, O, F, and Ti elements in DCMP (Fig. S6b), confirming that MXene forms stable and evenly distributed bonds with cellulose and CP-DES. These results provide further confirmation of the successful synthesis of a stable eutectogel network with enhanced structural properties.

To examine the temperature-dependent behavior of DCMP eutectogel, we performed variable temperature Fourier transform infrared (VT-FTIR) spectroscopy as the temperature was increased from 30 to 120 °C in 5 °C increments. As shown in Fig. 2c-e, with increasing temperature, the O–H stretching vibrations (3300–3400 cm^–^^1^) of cellulose and phytate exhibit progressive changes in both band shape and intensity. These changes indicate a gradual weakening of the hydrogen bonds formed between phytate, MXene, and cellulose/PAM [33]. As shown in Fig. 2c-e, with increasing temperature, the O–H stretching vibrations (3300–3400 cm^–^^1^) from cellulose and phytate demonstrate changes in both band shape and intensity. This change reflects the weakening of hydrogen bonds formed between phytate, MXene, and cellulose/PAM. As the increase in temperature, the hydrogen bonds in the system are progressively broken, reducing the constraints on the O–H bonds. Additionally, the peaks around 1300–1330 cm^–1^, corresponding to P–O–C stretching vibrations, exhibit decreasing intensity and slight shifts, further supporting the thermal instability of the coordination bonds between phytate and MXene. Similarly, the peaks around 1380–1500 cm^–^^1^, corresponding to C=O and C–N stretching vibrations, sharpen with rising temperature, indicating a thermally induced disruption of these bonds. These spectral changes reflect the dynamic nature of the intermolecular interactions within the eutectogel, which respond reversibly to temperature fluctuations.

The water states in the CMP and DCMP were analyzed using LF-NMR based on relaxation time differences (Fig. 2f), with shorter transversal relaxation time (*T*_2_) values indicating stronger water-binding affinity to the materials and reduced water activity, quantitatively distinguishing three water states: bound H_2_O (*T*_2_ = 0.1–4.0 ms, tightly associated), network H_2_O (*T*_2_ = 4–50 ms, structured immobile), and free H_2_O (*T*_2_ = 50–1000 ms, bulk-like) [34]. As shown in Fig. 2f, CMP mainly contains free water, while DCMP consists mostly of network water, indicating that the interaction between water molecules and DES forms a hydrogen bond network [35, 36]. This network effectively limits the contact between free water and MXene, thereby protecting the MXene structure from oxidation.

To assess the thermal stability of the eutectogels, TGA, derivative thermogravimetry (DTG), and DSC were performed. Thermogravimetric analysis reveals that the incorporation of MXene improves the thermal stability of the eutectogels, as evidenced by the lower mass loss rates of CMP and DCMP compared to pure cellulose (200–350 °C, Fig. 2g) [37]. The enhanced stability is further supported by the DTG data (Fig. 2h), with DCMP exhibiting a slower degradation rate than CMP in the range of 400–450 °C, underscoring the additional stabilizing effect of the DES. This improvement is attributed to the inherent stability of the CP-DES and its protective effect on cellulose. The enhanced thermal integrity is further confirmed by DSC, which shows a heat flow variation of 18 mW for CMP compared to a more stable 14 mW for DCMP (Fig. S7), indicating that the DES helps suppress phase transitions and ensures reliable performance in practical applications [38].

Molecular simulations elucidate the dynamic bonding process during eutectogel formation (Fig. 2i). The process begins with unbound components, followed by the initial formation of a stable CP-DES network through hydrogen bonds between phytic acid and choline chloride [39]. This network then interacts with and weakens the internal hydrogen bonding of cellulose. Subsequently, the introduction of MXene triggers a rapid surge in CP-DES-MXene hydrogen bonds at ~ 2500 ps that stabilizes, leading to a 49.8% increase in the number of hydrogen bonds (Fig. 2j). This key step is driven by strong interactions between the DES and MXene’s hydroxyl groups, with water molecules further facilitating the formation of a stabilizing hydrogen bonding network. Consequently, this robust interfacial bonding ensures uniform MXene dispersion and strong cellulose-MXene interactions, ultimately underpinning the superior mechanical strength and stability of the DCMP eutectogel.

### Multifunctional Properties of DCMP Eutectogel

**Fig. 3:**
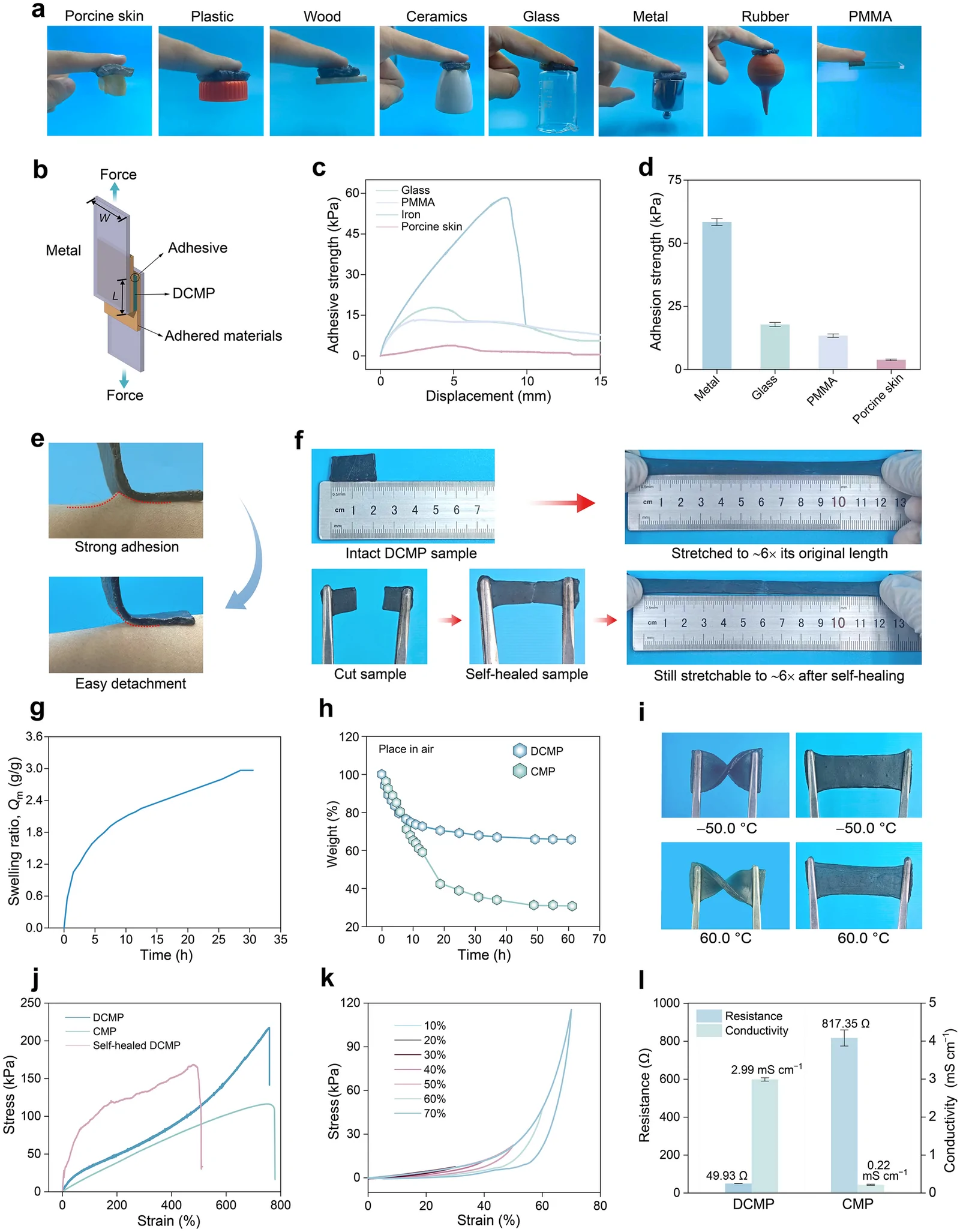
Adhesion, self-healing, swelling, stability, mechanical resilience, and conductivity of DCMP eutectogel. **a** Photographs of the DCMP eutectogel with excellent adhesion to different representative substrates. **b** Shear strength by the standard lap-shear test. **c** Adhesive strength curves of DCMP to various representative substrates. **d** Shear strength of the DCMP eutectogel to various representative substrates. **e** DCMP adheres to the surface of human skin and leaves no residual substances after being removed. **f** Self-healing performance test of DCMP. **g** Swelling ration curves of DCMP. **h** Water retention capacity of DCMP and CMP. **i** Stability test of eutectogels DCMP under extreme conditions. **j** Stress–strain curves of CMP, DCMP, and DCMP after healing. **k** Compression stress curves of DCMP under different strains. **l** Internal resistance and conductivity of CMP and DCMP

Strong adhesion is essential for eutectogels in wound dressing applications. The DCMP eutectogel conforms robustly to human fingers and diverse substrates without visible gaps, demonstrating broad-spectrum adhesion (Fig. 3a). Quantitative measurements on various materials reveal its highest adhesive strength on metal (58.4 kPa), with significant adhesion to glass (17.8 kPa), Poly(methyl methacrylate) (PMMA) (13.4 kPa), and porcine skin (3.8 kPa) (Fig. 3b-d). Importantly, the DCMP eutectogel adheres strongly yet can be cleanly removed from skin without residue (Fig. 3e). This optimal adhesion arises from hydrogen bonding and π–π interactions involving its hydroxyl, carboxyl, and aryl groups, combined with the inherent tackiness of the DES [40]. Medical gels often experience continuous external mechanical forces after adhesion, leading to potential deformation or damage [41]. To assess the self-healing capabilities of DCMP, we performed cutting tests and found that DCMP eutectogel rapidly self-heal within 20 s of being cut, maintaining stretchability without breaking. Even after 12 h, the material can stretch up to six times its original length without cracks, preserving its mechanical properties (Fig. 3f). In addition, the DCMP eutectogel before and after self-healing was observed under an optical microscope (Fig. S8). The originally wide cracks in the DCMP completely closed and disappeared after self-healing. This self-healing ability is attributed to the dynamic hydrogen bonding interactions within the eutectogels.

An ideal hemostatic material must combine strong adhesion with liquid absorption and swelling capability [42]. The DCMP eutectogel exhibits this critical attribute, rapidly absorbing water and swelling within the first 10 h before the swelling ratio (*Q*_*m*_) equilibrates at approximately 3.0 after 30 h (Fig. 3g). This kinetics is driven by its hydrophilic groups, which effectively bind water and expand the network. The material’s hydrophilicity is further corroborated by a water contact angle of 69.18° (Fig. S9), a property that facilitates efficient blood absorption [32].

Water retention is also crucial for the long-term stability of eutectogels. As shown in Fig. 3h, CMP lost 69.18% of their weight in 20 h, causing shrinkage and structural loss. In contrast, DCMP eutectogel only loses 34% of their weight after 60 h at room temperature, maintaining their structure. This improved retention is due to the stable hydrogen bond network between cellulose and MXene, which effectively locks the CP-DES solvent. To simulate extreme conditions, we freeze-dried the eutectogels at − 50 °C for 48 h and baked them at 60 °C for 24 h. Remarkably, the DCMP eutectogel retains their toughness and flexibility, showing good resistance to both freezing and high temperatures (Fig. 3i). This stability is primarily attributed to the addition of PA from DES, which prevents water crystallization, and the strong MXene-cellulose network, which enhances the material’s thermal resistance [43].

Mechanical properties are essential for evaluating the structural stability and practical applications of eutectogels [44]. As shown in Fig. 3j, CMP eutectogels have a Young’s modulus of 114 kPa and an elongation at break of 779%. Upon adding CP-DES, the Young’s modulus of DCMP increases to 217 kPa, while the elongation at break remains relatively unchanged (757%), suggesting that CP-DES synergistically enhances the interaction between MXene and cellulose. In compression tests, DCMP eutectogel is able to withstand a stress of 115 kPa at 70% strain (Fig. 3k), with a well-closed compression-rebound curve, indicating good resilience. This is due to the robust network binding within the eutectogels, contributing to their excellent mechanical properties. To further evaluate its fatigue resistance, DCMP was subjected to 60 continuous loading–unloading cycles (Fig. S10). After initial stress softening, the subsequent hysteresis loops overlapped nearly perfectly, confirming its excellent anti-fatigue properties and long-term mechanical robustness for practical applications [45, 46]. Furthermore, compared with previously reported eutectogels (Table S2), the DCMP eutectogel exhibits excellent mechanical performance in both tensile stress–strain and compressive stress–strain behaviors.

Furthermore, MXene and CP-DES impart DCMP eutectogel with remarkable electrical conductivity, which is essential for their use as sensors. The internal resistance of DCMP is 49.93 Ω, significantly lower than that of CMP (817.35 Ω), with a conductivity of 2.99 mS cm^–1^ (Fig. 3l). Notably, even after the self-healing, the DCMP still maintains a low internal resistance and a high conductivity (Fig. S11), demonstrating its excellent recovery capability of ionic transport after self-healing [47]. The presence of PA in CP-DES enhances the conductivity within the DCMP network, while MXene facilitates electron transfer through the cellulose channels. A touch screen experiment was conducted by wrapping DCMP around a wooden manipulator, which was unable to interact with the screen until the eutectogels were applied. When adhered to a manipulator, the DCMP eutectogel functionally substitutes for a human finger by enabling precise touch identification (Fig. S12). This demonstrates its significant potential for integration into touch‐sensitive interfaces and skin‐conformal sensors, operating effectively without impeding natural manual functions. The DCMP eutectogel demonstrates versatile motion-sensing capability across various articulation sites (Fig. S13a). When attached to the Adam’s apple, fingers, elbows, and knees, it generates distinct and repeatable signals in response to speaking (“hello”), swallowing and coughing (Fig. S13b-d), confirming its high sensitivity and reliability. The DCMP eutectogel could distinguish different motion signals, indicating their potential for human activity detection. The sensor exhibits substantial relative resistance changes (∆*R/R*_*0*_) of 120%, 200%, and 500% upon the bending of finger joints, elbows, and knees, respectively (Fig. S13e-g). These results confirm the sensitivity and excellent adhesion performance of DCMP eutectogel [48]. The DCMP eutectogel was further evaluated for handwriting recognition under a PET film (Fig. S13h). It successfully distinguishes the word “TUST” with unique electrical signatures for each letter and shows consistent signal responses for repeated characters (Fig. S13i), demonstrating its capability for repeatable touch sensing. Moreover, writing numbers “1–9” on the DCMP eutectogel surface generates distinct signal variations for each digit (Fig. S13j), demonstrating its high resolution in discriminating between subtle stress and strain differences.

### Photothermoelectric Conversion Performance of DCMP Eutectogel

**Fig. 4:**
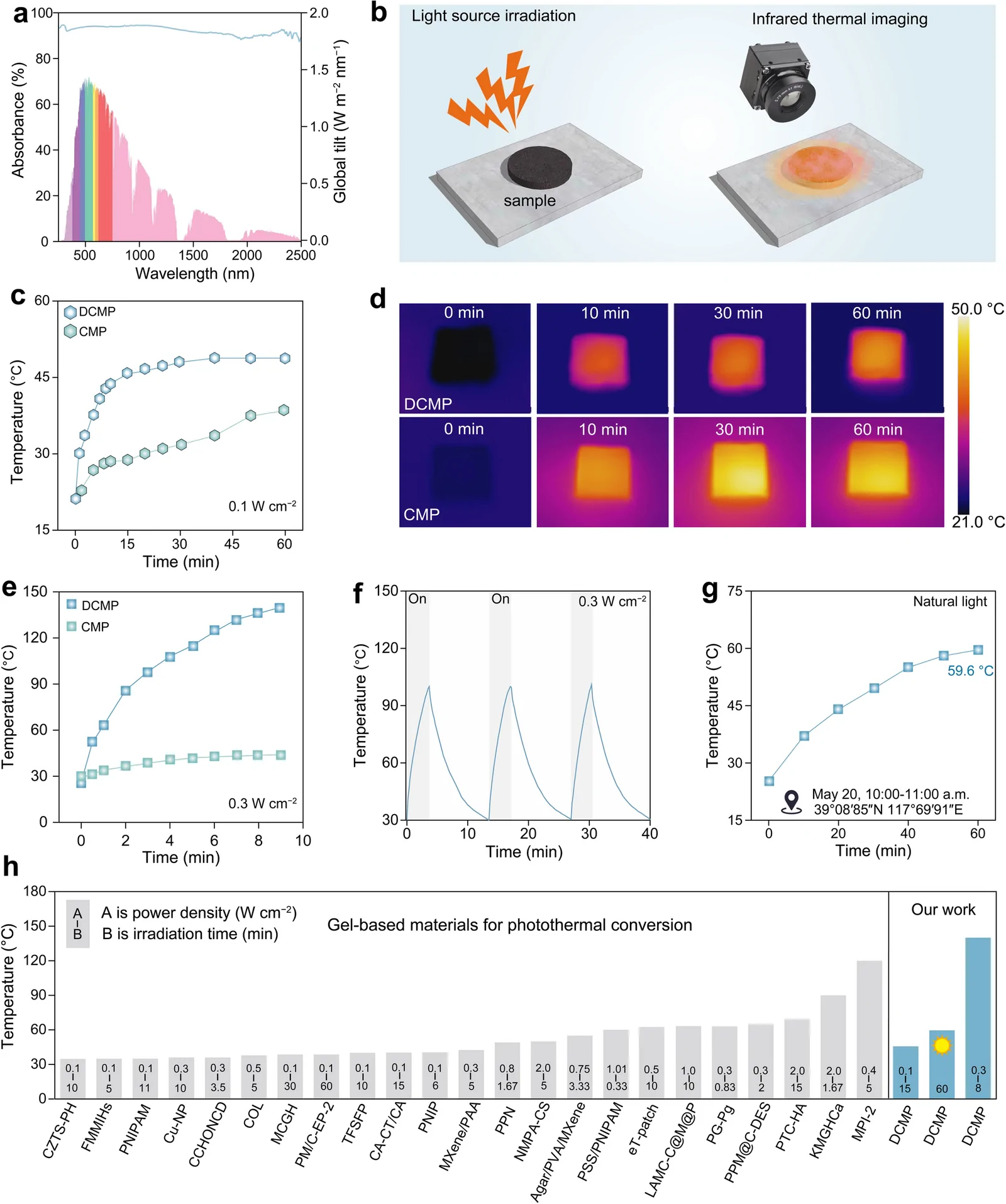
Photothermal conversion performance of DCMP eutectogel. **a** Absorption of DCMP at different wavelengths. **b** Schematic diagram of the photothermal conversion of DCMP under simulated sun irradiation. **c** Temperature changes and **d** thermal imaging images of CMP and DCMP under simulated sunlight with radiation intensity of 0.1 W cm^–2^. **e** DCMP temperature change test under simulated sunlight with radiation intensity of 0.3 W cm^–2^. **f** Photothermal cycle test of DCMP under simulated sunlight with radiation intensity of 0.3 W cm^–2^. **g** Temperature change curve of DCMP under natural light. **h** Comparison of DCMP with other gel-based photothermal conversion materials

The optical characteristics of the eutectogels are directly associated with their photothermal conversion performance. We initially evaluated the optical behavior of DCMP (Figs. 4a and S14). DCMP displays near-complete light blockage, with transmittance close to 0% and very low reflectance across the full spectral range, suggesting that over 90% of incident light is absorbed by the eutectogels. This pronounced absorption is ascribed to the LSPR on MXene surfaces, arising from the collective oscillation of free electrons driven by the alternating electric field of incident light [49]. These findings confirm that MXene remains free from oxidation in the eutectogel system, a stability attributed to the presence of DES.

To evaluate the photothermal conversion capacity of DCMP eutectogel, we irradiated the surface of the material with a xenon lamp and recorded its temperature using a thermal imager (Fig. 4b). Under a light intensity of 0.1 W cm^–^^2^, the CMP eutectogel reaches 38.5 °C after 60 min, whereas the DCMP eutectogel heats rapidly to 40.9 °C within 7 min and stabilizes at 49.6 °C by 40 min (Fig. 4c, d). This performance enhancement results from the presence of DES, which intensifies the LSPR effect of MXene and thereby elevates the photothermal conversion efficiency of DCMP. The photothermal conversion efficiency (PCE), calculated using Eqs. S2 and S3, reaches a notable value of 67.9% for DCMP. For a more comprehensive evaluation, we increased the xenon lamp’s irradiation intensity to 0.3 W cm^–2^ and tested the photothermal cycling performance of the DCMP eutectogel. Under this increased light intensity, the temperature of DCMP rapidly rises, reaching 140 °C within 9 min, while CMP only reaches 44 °C (Figs. 4e and S15). To assess the cycling stability, we subject the sample to on–off cycles of the xenon lamp. DCMP quickly heats to 100 °C in each cycle and maintains this performance over three cycles (Fig. 4f), demonstrating excellent photothermal stability. To evaluate its potential for practical application, we test the photothermal performance of the DCMP eutectogel under natural sunlight. The experiment was conducted from 10:00 to 11:00 a.m. on May 20, 2024, in Tianjin, China (39° 08′ 85″ N, 117° 69′ 91″ E). Under these conditions, the DCMP eutectogel exhibits a rapid and linear increase in surface temperature, reaching 61 °C after 60 min of exposure (Fig. 4g). This result confirms the material’s practicality for practical application. Furthermore, a comparison with other gel-based materials from the literature shows that DCMP stands out due to its excellent photothermal conversion, low energy consumption, and fast light response (Fig. 4h and Table S3).

**Fig. 5:**
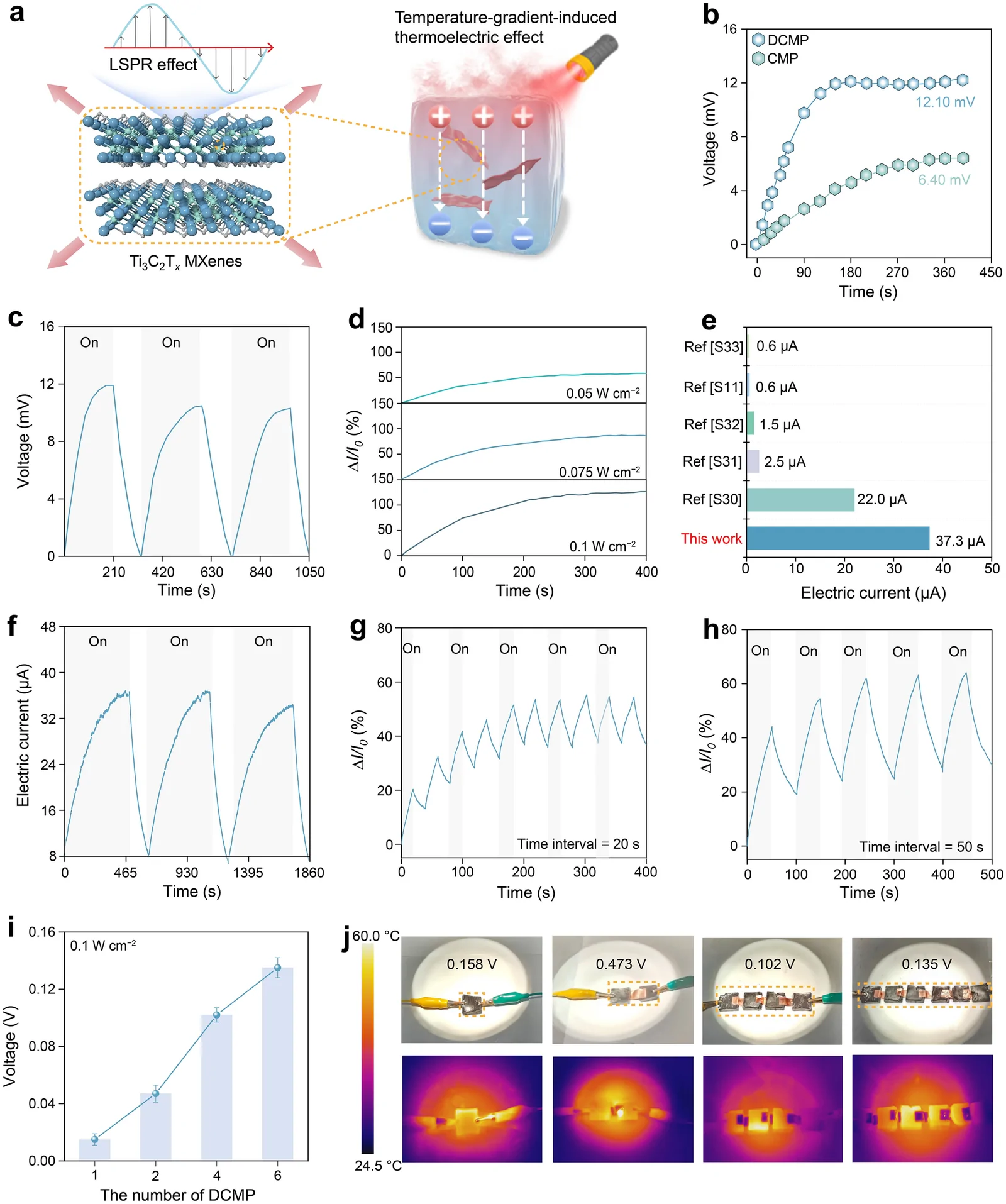
Thermoelectric conversion performance of DCMP eutectogel. **a** Schematic diagram of the photothermoelectric in DCMP eutectogel. **b** Voltage changes of CMP and DCMP with radiation intensity of 0.1 W cm^–2^. **c** Thermoelectric cycle test of DCMP with radiation intensity of 0.1 W cm^–2^. **d** Voltage changes of DCMP with different radiation intensity. **e** Comparison of DCMP with other gel-based thermoelectric conversion materials. **f** Thermoelectric cycle test of DCMP with radiation intensity of 0.1 W cm^–2^. Thermoelectric cycle test of DCMP at **g** 20 s and **h** 50 intervals with radiation intensity of 0.1 W cm^–2^. **i** Photothermoelectric conversion performance and **j** thermal imaging images of multiple DCMP eutectogel under radiation intensity of 0.1 W cm.^–2^

Photo-thermo-electric conversion technology is a promising approach for utilizing solar energy, employing photothermal materials as the hot-side interface of thermoelectric modules to leverage the Seebeck effect (Fig. 5a) [50]. Under 0.1 W cm^–^^2^ simulated sunlight, the power generation measurements reveal a distinct difference: CMP shows a slow voltage increase to a maximum of 6.40 mV, while DCMP rapidly reaches 12.10 mV in 150 s (Fig. 5b). Furthermore, under an irradiation power density of 0.3 W cm^–^^2^, the DCMP reached a stable voltage of approximately 33.5 mV after 200 s (Fig. S16). This response is highly reversible, as the voltage promptly returns to baseline upon turning off the light. Furthermore, DCMP maintains stable and repeatable voltage output over three consecutive cycles (Fig. 5c), confirming its robust performance. After exposure to simulated sunlight for 300 s at intensities of 0.05, 0.075, and 0.1 W cm^–2^, the current generated by DCMP is increased by 57%, 86%, and 124%, respectively, compared to the initial current (Fig. 5d). DCMP exhibits superior performance with a current output of 37.3 μA at 0.1 W cm^–2^, outperforming existing systems documented in the previous literatures (Fig. 5e and Table S4). The material also shows a highly reversible response, as the current rapidly recovers to its initial level within 150 s after the light is turned off (Fig. 5f). This combination of high sensitivity and reliable repeatability demonstrates that DCMP possesses the essential characteristics required for advanced thermoelectric applications.

To further investigate the thermoelectric cycling performance, we conducted tests with fixed turn-on and turn-off times of 20–50 s, respectively. As shown in Fig. 5g, h, the current of DCMP rapidly increases upon illumination and continues to rise for a period even after the xenon lamp is turned off, indicating a sustained current generation. This behavior further confirms the superior thermoelectric performance and cycling capability of DCMP.

To this end, we connected multiple DCMP eutectogel in series to enhance power generation. Under illumination, the integrated system maintains excellent photothermal performance, with surface temperatures reaching 50–60 °C (Fig. 5i, j). Notably, a series connection of six DCMP eutectogel yields a self-generated voltage of 0.135 V after a period of irradiation. This result demonstrates that a series configuration leads to a higher voltage output, highlighting the potential of these materials for practical energy harvesting applications.

### Biocompatibility and In Vitro Hemostatic Performance of DCMP Eutectogel

**Fig. 6:**
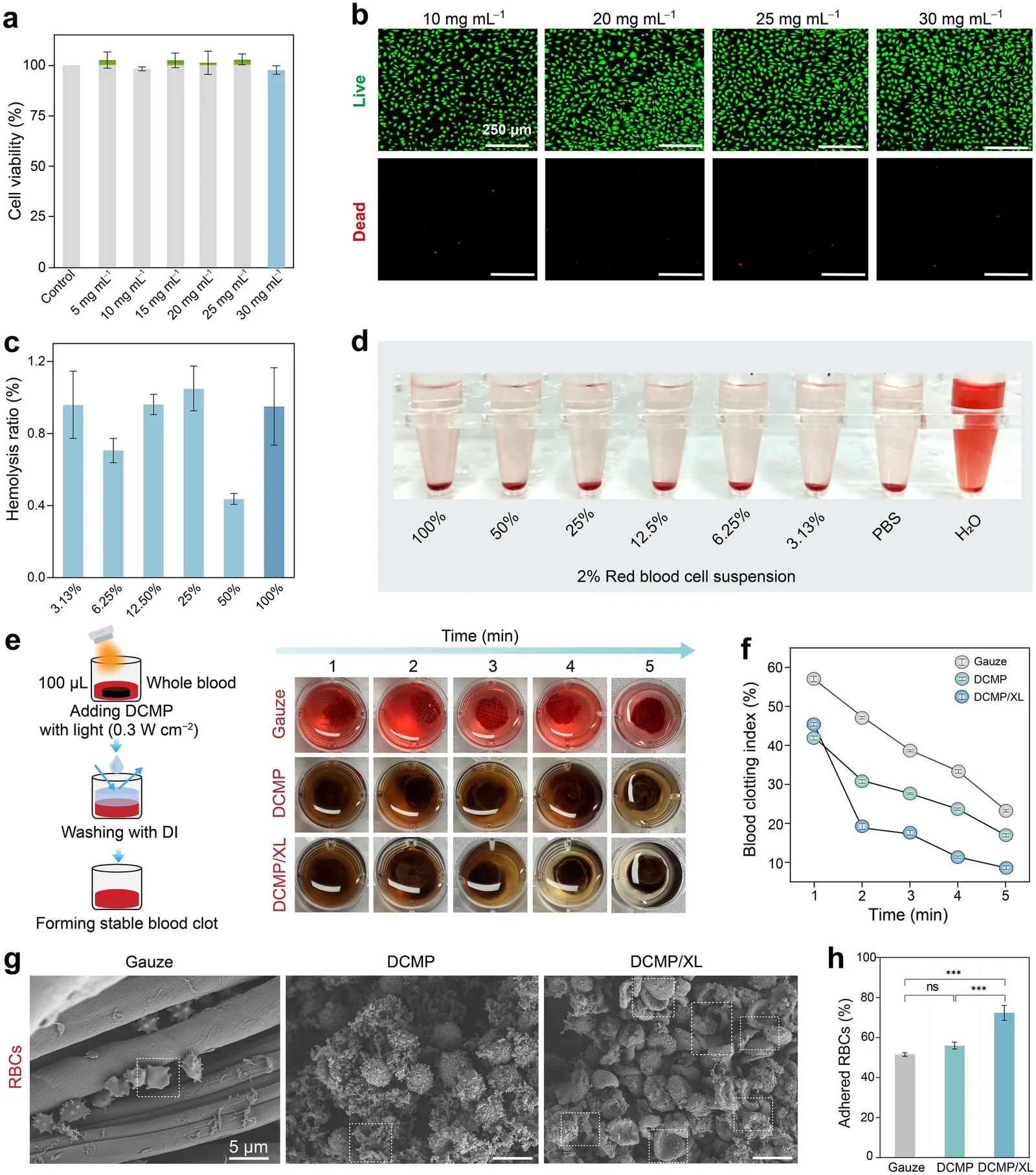
Cytocompatibility, hemocompatibility and in vitro hemostatic performance of DCMP eutectogel. **a** Cytocompatibility of L929 cells treated with extracts from different of DCMP over 24 h, *n* = 3. **b** Live/dead staining images of DCMP. **c** Hemolysis ratio of DCMP, *n* = 3. **d** Photographs from hemolytic activity assay of DCMP. **e** Schematic illustration and dynamic clotting tests of blood of the guaze, DCMP, and DCMP with radiation intensity of 0.3 W cm^–2^,* n* = 3. **f** BCI (%) of gauze, DCMP, and DCMP with radiation intensity of 0.3 W cm^–2^,* n* = 3. **g** SEM scanning of adhesion behavior of gauze, DCMP, and DCMP with radiation intensity of 0.3 W cm^–2^ to blood cells. **h** Adhered RBCs of gauze, DCMP, and DCMP with radiation intensity of 0.3 W cm^–2^,* n* = 3. (Data is presented as mean ± S.D.) (ns: not significant; *p < 0.05, **p < 0.01, ***p < 0.001, and ****p < 0.0001)

Biocompatibility is a critical requirement for the in vivo application of eutectogels [51]. We assess the cytocompatibility of DCMP using live/dead staining and a Cell Counting Kit-8 (CCK-8) assay. As shown in Fig. 6a, L929 fibroblasts are co-cultured with extracts from six different concentrations of DCMP for 24 h. The cell viability remains above 97% across all groups, indicating excellent cytocompatibility. These findings are corroborated by live/dead staining (Fig. 6b), which shows a majority of viable, spindle-shaped cells stained green, with only a minimal presence of red fluorescent cells [52]. Next, the hemocompatibility of DCMP eutectogel is evaluated through an in vitro hemolysis assay. Quantitative analysis shows that the hemolysis ratio for all DCMP groups remains below 5%, which meets the international standard for biomedical materials (Fig. 6c) [53]. As shown in Fig. 6d, the H_2_O-positive control exhibits complete hemolysis, whereas the color of the DCMP extracts is similar to the pale red of the PBS-negative control (Fig. 6d), indicating minimal red blood cell disruption. These results confirm the high hemocompatibility of DCMP eutectogel, supporting its potential for in vivo application.

A critical consideration for applying photothermoelectric conversion to hemostasis is achieving rapidly the requisite temperature and current while avoiding tissue damage [54]. Balancing rapid photoresponse with biological safety, we selected a light intensity of 0.3 W cm^–^^2^ for all subsequent coagulation and hemostasis experiments. We assessed the hemostatic performance of DCMP using the blood clotting index (BCI, %) (Fig. 6e) [55]. The result reveals a distinct contrast, i.e., the gauze group remains bright red over 1–5 min, signaling inefficient coagulation and substantial red blood cells (RBCs) residue, whereas DCMP demonstrates a clear coagulation effect. This capability likely stems from the MXene properties, which facilitate clotting through their large specific surface area, oxygen-containing functional groups, and net negative charge [56]. Notably, DCMP under 0.3 W cm^–2^ xenon lamp irradiation (DCMP/XL) exhibits the lightest color, suggesting enhanced blood coagulation driven by its photothermoelectric properties. Quantitative analysis reveals that DCMP/XL exhibits the lowest BCI (8.62%) at 5 min, outperforming both the gauze and non-irradiated DCMP groups (Fig. 6f). A lower BCI value indicates superior procoagulant properties, confirming the effective blood coagulation ability of DCMP with photothermoelectric capabilities [57].

The underlying mechanism for improved clotting is probed by analyzing the adhesion of RBCs and platelets. Direct visualization via SEM shows dense aggregates of these cells on DCMP/XL (Fig. 6g), far exceeding the levels seen in other groups. Quantification of RBC adhesion (Fig. 6h) confirms a significant enhancement, with rates of 51.48% (gauze), 55.94% (DCMP), and 77.2% (DCMP/XL). This marked increase in cell adhesion under photothermoelectric stimulation directly explains the superior coagulation performance of DCMP/XL, underscoring its potential as an effective hemostatic agent.

### In Vivo Hemostatic Performance of DCMP Eutectogel

**Fig. 7:**
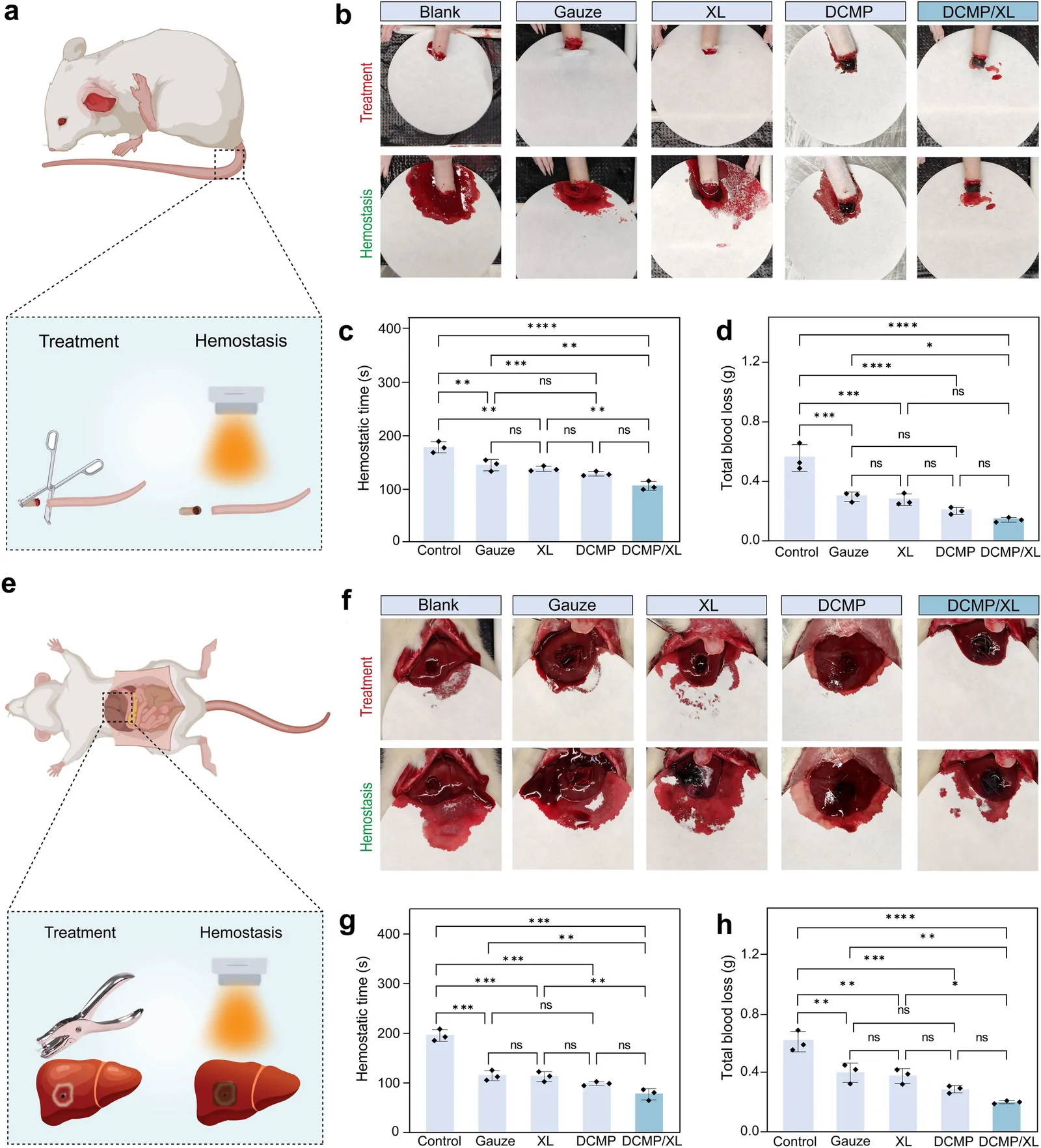
In vivo hemostatic performance of the DCMP eutectogel. **a** Schematic illustration of the tail amputation hemorrhage model in rats treated by eutectogels. **b** Photographs of the tail amputation hemorrhage model in rats treated by guaze, xenon lamp, DCMP, and DCMP/XL with radiation intensity of 0.3 W cm^–2^,* n* = 3. **c** Hemostatic time and **d** quantification of total blood loss of the tail amputation hemorrhage model in rats treated by guaze, xenon lamp, DCMP, and DCMP/XL with radiation intensity of 0.3 W cm^–2^,* n* = 3. **e** Schematic illustration of the liver perforation hemorrhage model in rats treated by DCMP with photothermoelectric. **f** Photographs of the liver perforation hemorrhage model in rats treated by guaze, xenon lamp, DCMP, and DCMP/XL with radiation intensity of 0.3 W cm^–2^,* n* = 3. **g** Hemostatic time and **h** quantification of total blood loss of the liver perforation hemorrhage model in rats treated by guaze, xenon lamp, DCMP, and DCMP/XL with radiation intensity of 0.3 W cm^–2^,* n* = 3. (Data is presented as mean ± S.D.) (ns: not significant; *p < 0.05, **p < 0.01, ***p < 0.001, and ****p < 0.0001)

The hemostatic efficacy of the DCMP eutectogel was evaluated in two distinct rat models: tail amputation and liver perforation. These models simulate acute bleeding from trunk and visceral organs, thus allowing a comprehensive assessment of its performance [58]. In the tail amputation model, a clear reduction in bleeding volume is observed in the DCMP/XL-treated groups (Fig. 7a, b). The DCMP/XL group demonstrates superior hemostatic efficacy, achieving complete hemostasis in only 104.7 s with a minimal blood loss of 0.15 g. In contrast, all control groups require significantly longer times and exhibit greater bleeding volumes: control (177 s, 0.56 g), gauze (143.7 s, 0.30 g), xenon light only (136.7 s, 0.28 g), and DCMP only (127.3 s, 0.21 g) (Fig. 7c, d and Table S5). Similarly, in the liver perforation model (Fig. 7e, f), the DCMP/XL group exhibited superior performance, achieving the most rapid hemostasis (80.7 s) and lowest blood loss (0.21 g). This result stands in clear contrast to the other groups: control (199 s, 0.61 g), gauze (118 s, 0.40 g), xenon light only (116.3 s, 0.38 g), and DCMP only (102 s, 0.29 g) (Fig. 7g, h and Table S6). It is noteworthy that, in both hemorrhage models, the DCMP eutectogel with photothermoelectric conversion demonstrates significantly reduced blood loss and shorter hemostasis times than all other groups. This enhanced performance stems from its ability to convert light into localized heat. The generated heat promotes the accumulation of platelets and coagulation factors at the wound site, thereby accelerating the coagulation cascade [59]. Additionally, the localized thermal energy from the photothermal effect induces the coagulation of proteins and clotting factors, forming a protective clot that seals the wound, a process termed thermal coagulation [60]. This thermal action is complemented by the weak electrical current generated via the thermoelectric effect, which contributes to hemostasis by inducing local vasoconstriction [61].

These results collectively highlight the rapid hemostatic capabilities of DCMP eutectogel, demonstrating significantly reduced blood loss and hemostasis time in vivo. The synergistic effect of photothermal conversion, along with the eutectogel’s intrinsic adhesive and blood-absorptive properties, enhances hemostasis. During the hemostasis process, conventional gauze, light irradiation alone, or DCMP without light irradiation are insufficient to rapidly control bleeding. In contrast, DCMP eutectogel achieves synergistic hemostasis through the combined effects of photothermal heat generation, which quickly aggregates red blood cells, and the weak electrical current, which promotes vasoconstriction. The synergistic action of these dual mechanisms accounts for the exceptional hemostatic efficacy of the DCMP eutectogel, thereby establishing their promise as a versatile material for trauma care and surgical applications. In brief, compared to commercial hemostats such as fibrin glues (expensive, risk of viral transmission) or QuikClot/Celox (specialized synthetic products), DCMP offers significant cost advantages. Its matrix uses abundant, low-cost bio-based cellulose, while choline chloride and phytic acid are inexpensive bulk chemicals. Although MXene has a higher unit cost, its mass fraction in the eutectogel is extremely low, exerting negligible impact on overall cost. Moreover, the DES-mediated one-pot synthesis under mild room temperature conditions avoids toxic solvents and complex purification, enabling scalable roll-to-roll production.

## Conclusion

In this work, we demonstrate that integrating MXene into a DES-mediated cellulose–polyacrylamide network yields a multifunctional eutectogel that couples robust adhesion, efficient fast liquid uptake and swelling, and multimodal photothermoelectric conversion for rapid hemostasis. Unlike conventional gauze or passive hemostatic dressings, DCMP exploits light-driven local heating and Seebeck-induced microcurrents to actively accelerate clot formation, achieving markedly reduced blood loss and hemostasis time in both rat tail amputation and liver perforation models compared with all control groups. Enhanced RBC and platelet adhesion under irradiation further corroborates the synergistic contribution of photothermal and thermoelectric effects to coagulation. In this strategy, the CP-DES plays a central role in reconciling mechanical integrity with functional performance. By in situ dissociating cellulose and intercalating MXene, it produces a homogeneous, tough, and self-healable network with high conductivity, excellent water retention, and anti-freezing capability down to − 50 °C, while its antioxidative microenvironment effectively suppresses MXene oxidation and preserves its LSPR-mediated photothermal response over repeated cycles and even natural sunlight exposure. These structural and physicochemical features underpin the stable photothermoelectric output and sustained hemostatic efficacy of DCMP. Moving forward, optimizing light delivery and dose in deep or irregular wounds, tailoring degradation behavior, and validating performance in larger-animal and clinically relevant bleeding models are crucial to realize the potential of this photothermoelectric eutectogel platform for practical trauma care and surgical hemostasis.

## Supplementary Information

Below is the link to the electronic supplementary material.

Supplementary file1 (DOCX 10168 KB)
