# Supplementary material for: Unveiling Photo-Thermal-Electrical Performance in Robust, Self-Healing, and Anti-Freezing Cellulose-MXene Eutectogels for Advanced Hemostasis
Source: Nanomicro Lett. 2026 Jul 20;18:444. doi: 10.1007/s40820-026-02283-9 (PMC13385548; doi:10.1007/s40820-026-02283-9)
Supplement: Supplementary file 1 — Supplementary file1 (DOCX 10168 KB) [file 40820_2026_2283_MOESM1_ESM.docx]

Supporting Information for

**Unveiling Photo-Thermal-Electrical Performance in Robust, Self-Healing, and Anti-Freezing Cellulose-MXene Eutectogels for Advanced Hemostasis**

Chuang Jiang ^1#^, Hengli Ning ^1#^, Wei Liu ^1,*^, Zhikun Li ^1^, Huiwu Zhu ^1^, Long Li ^1,*^, Qingxi Hou ^1^, Chaoji Chen ^2,*^, Bowen Cheng ^1,*^

^1^ State Key Laboratory of Bio-Based Fiber Materials, China Textile Industry Key Laboratory of High-Performance Fibers Wet-Laid Nonwoven Materials, Tianjin Key Laboratory of Pulp & PaperTianjin University of Science and Technology, Tianjin, 300457, People’s Republic of China

^2^ Hubei Biomass-Resource Chemistry and Environmental Biotechnology Key Laboratory, Hubei Provincial Engineering Research Center of Emerging Functional Coating Materials, School of Resource and Environmental Sciences, Wuhan University, Wuhan 430079, P. R. China

#Hengli Ning and Chuang Jiang contributed equally to this work.

*Corresponding author. E-mail: [weiliu@tust.edu.cn](mailto:weiliu@tust.edu.cn) (Wei Liu); [lilong@tust.edu.cn](mailto:lilong@tust.edu.cn) (Long Li); [chenchaojili@whu.edu.cn](mailto:chenchaojili@whu.edu.cn) (Chaoji Chen); [bowenc15@tust.edu.cn](mailto:bowenc15@tust.edu.cn) (Bowen Cheng)

**S1 Materials and methods**

**S1.1 Materials**

The cellulose fibers were provided by a paper company in Shandong province, China. Choline chloride (ChCl, 99%) and phytic acid (PA, 50%) were purchased from Shanghai Macklin Biochemical Technology Co., Ltd. Acrylamide (AM, 99%) was purchased from Tianjin Damao Chemical Reagent Factory. Ammonium persulfate (APS, 99%) was purchased from Tianjin Jindong Tianzheng Fine Chemical Reagent Factory. N, N'-methylene bisacrylamide (MBA, 99%) was purchased from Shanghai Bider Pharmaceutical Technology Co., Ltd. Dulbecco’s Modified Eagle Medium (DMEM) was purchased from XP Biomed Ltd. Fetal Bovine Serum was purchased from ExCell Bio Co., Ltd. L929 cells (a mouse fibroblast cell line) were purchased from the Cell Bank of the Chinese Academy of Sciences (Shanghai, China). The Live/Dead Cell Viability/Cytotoxicity Assay Kit and the Cell Counting Kit-8 (CCK-8) were purchased from Jiangsu KeyGen Biotech Co., Ltd. Phosphate-buffered saline (PBS) buffer and Ethylenediaminetetraacetic acid (EDTA) anticoagulant were purchased from Beijing Solarbio Science & Technology Co., Ltd. All other chemicals were of analytical grade.

**S1.2 Preparation of Layered MXene**

Lithium fluoride (LiF) was dissolved in a hydrochloric acid (HCl) solution. A quantified amount of Ti_3_AlC_2_ was then gradually introduced into this solution under continuous stirring for 24 h. Following the reaction, the solid product was isolated via centrifugation. To purify the product, it was sequentially washed with HCl three times to eliminate any remaining LiF, followed by thorough washing with distilled water until a pH value above 6 was achieved. Subsequently, the precipitate was vacuum-dried, taken out and ground into powder, thus obtaining layered Ti_3_C_2_T_x_ MXene powder.

**S1.3 Pretreatment of Cellulose Fibers**

The pulp fibers were first manually torn into approximately 1 cm × 1 cm pieces. These pieces were then ground using a pulverizer. The resulting fibrous material was sieved, and fibers retained on 40–60 mesh sieves were collected for subsequent use.

**S1.4 Characterizations of the DCMP eutectogels**

To evaluate the long-term anti-oxidation capability of the solvent system, the MXene/CP-DES dispersion was monitored for a long time. The dispersion was placed in a transparent glass vial (leaving an air head-space) and stored under ambient laboratory conditions. The environmental temperature was maintained at approximately 25 ± 2 °C with a relative humidity of 45~55%, and the samples were exposed to standard ambient indoor lighting. The chemical structures of samples were analyzed by FTIR spectrometer (Model 650, Tianjin Gangdong Sci. & Tech. Development Co., Ltd., China) within 4000 to 800 cm^−1^. The internal bonding analysis of the samples from 30 °C to 120 °C was carried out by variable temperature Fourier transform infrared spectroscopy (VT-FTIR, 32 times of scanning, resolution of 4 cm^−1^, invenio-s, Bruker, Germany). Raman mapping was used to analyze the composition distribution of the samples, using a ThermoFisher Scientific DxR instrument (USA). The water states in the samples were analyzed by low-field nuclear magnetic resonance (LF-NMR) (NMI20-015V-1, Niumag, China) based on relaxation time differences. The chemical component and interlamellar spacing of MXene was determined by an X-ray diffraction (XRD) (D8 Advance, Bruker-AXS, Germany) at a scan rate of 5 ° min^−1^.

The surface morphology and the cross-sectional morphology of samples were analyzed with SEM (JSM-IT 300LV, Japan), and further determined with software of Nano Measurer 1.2. Thermal stabilities of eutectogels were analyzed using TGA (Q50, TA, USA) from room temperature to 800 ℃ under N_2_ atmosphere. The thermal properties of the eutectogels were analyzed by a differential scanning calorimeter (DSC 8000, PerkinElmer, Inc., USA) from −60 to 200 ℃ at a heating rate of 10 ℃ min^−1^. The water retention of the eutectogels was evaluated by monitoring their weight loss after exposure to a controlled temperature and humidity environment.

The tensile and compressive strengths of the samples were tested using a universal testing machine (LE5106, Shanghai Lishi Co., Ltd., China). For tensile property testing, the mixed liquid was poured into a mold to form a dumbbell-shaped specimen (30.0 mm × 10.0 mm × 1.0 mm), and stress-strain testing was conducted at a rate of 20 mm min^−1^. For compression property testing, the mixed liquid was poured into a mold to polymerize and form a cylinder with a diameter of 33.0 mm and a height of 15.0 mm. The strain range was set to 10–70%, and stress–strain testing was performed at a compression rate of 20 mm min^−1^. The DCMP eutectogel (1 cm × 2 cm) was cut in half lengthwise, and the two halves were then allowed to bond for a specified period to complete the self-healing process. The DCMP eutectogel (2 cm × 4 cm) was stored at −50 °C for 12 hours, followed by twisting and tensile tests. The transmittance and reflectance of the eutectogels were characterized employing a UV-Vis-NIR spectrophotometer (UV 3600 Plus, Shimadzu, Japan) over 200–2600 nm, with a scan speed of 2 nm min^−1^, a slit width of 20 nm, and a threshold setting of 0.1.

The electrical signals of the eutectogels under different stimuli were recorded using a Keithley 2400 SourceMeter (Keithley, USA). The detection of human motions including graphomotor, body, and throat movements was performed with a volunteer who was also an author of this study (Chuang Jiang, male, 28 years old). The study protocol conformed to the ethical guidelines of the Declaration of Helsinki. Resistance and photoelectric conversion performance of eutectogels was analyzed with a multimeter (UNI-T, UT33A+). The photothermal conversion properties of the eutectogels were analyzed by monitoring their temperature changes using an infrared thermal imager (HM-TPH23-3AQFW, Hangzhou Hikmicro Sensing Technology Co., Ltd., China).

**S1.5 Water retention tests**

The water retention capacity was evaluated by subjecting test samples to controlled temperature and humidity conditions. Specifically, weight changes were recorded periodically over a 3-day period using an electronic scale. The retention ratio was then calculated according to the standard formula (S1):

$\text{WR}\text{=}\frac{\text{W}\text{i}}{\text{W}\text{0}}\text{×}\text{10}\text{0}\text{\%}$ (S1)

where *W_i_* and *W*_0_ represent the weight of the sample at time = *i* and 0, respectively.

**S1.6 Calculation of photothermal conversion efficiency (PCE)**

Specifically, PCE is defined as the ratio of the net heat output produced by the DCMP eutectogel sample to the total energy of the incident light irradiated onto the sample surface, which was calculated using the following equation (S2):

$\text{PCE=}\frac{\text{Q}_{\text{total}}\text{-}\text{Q}_{\text{blank}}}{\text{I∙S}}\text{×}\text{100\%}$  (S2)

where *Q_total_* is the total heat generated by the sample under irradiation, *Q_blank_* is the heat loss from the empty quartz container, *I* is the incident light intensity (0.1 W cm^–2^), and *S* is the effective irradiated area (1.0 cm^2^). A thermocouple (precision: ± 0.1 °C) was attached to the sample surface to record real-time temperature changes over 300 s. An empty quartz container (same size as the sample holder) was used for the blank control experiment. The total incident energy (*E*) over a duration time (*t*) is given by *E* = *I* · *S* · *t*, where *I* is the light intensity and *S* is the irradiated area. The calculated incident energy is 30 J when *t* is 300 s.

The heat output (Q) of test sample was determined based on the observed temperature change (Δ*T*) and its heat capacity, according to the relevant thermodynamic equation (S3):

$\text{Q=m∙}\text{C}_{\text{p}}\text{∙}\text{∆}\text{T}$ (S3)

where *m* is mass of test sample (0.5214 g), *C_p_* is specific heat capacity (*C_p_* = 1.8 J·g^–1^·K^–1^ for DCMP eutectogels), Δ*T* represents the temperature rise measured after 300 s of irradiation (Δ*T* = 21.7 K).

**S1.7 Calculation and simulation**

The partial charges of cellulose, PA (phytic acid), and ChCl molecules were calculated using the Gaussian 16 code with the 6‑311G(d,p) basis set. The general Amber force field (GAFF) was used to parameterize all atoms, including bond parameters, angle parameters, and dihedral angles. The interaction between MXene and different molecules was studied by molecular dynamics (MD) simulations, which were performed using the GROMACS 2021 software package. The steepest descent method was applied to minimize the initial energy of each system with a force tolerance of 1 kJ mol^−1^ nm^−1^ and a maximum step size of 0.002 ps before MD calculations. Periodic boundary conditions were imposed in all three directions. The leapfrog algorithm was used to integrate Newton's equations of motion. MD simulations were carried out in an NPT ensemble, and the simulation time was 20 ns.

**S1.8** **Cytotoxicity test**

L929 cells in logarithmic phase were seeded at densities of 4×10^3^ cells/well in 96-well plates and 1×10^5^ cells/dish in confocal dishes. The outer wells of the 96-well plate were filled with 100 µL sterile PBS to minimize evaporation. Subsequently, all cultures were incubated overnight at 37 °C in a 5% CO_2_ atmosphere. After 24 h of incubation, cells were treated for an additional 24 h with either gel extracts at various concentrations (5, 10, 15, 20, 25, and 30 mg L^–1^) or with complete medium alone (control). The gel extracts were prepared by autoclaving the gels, followed by extraction in complete medium for 24 h at a stock concentration of 60 mg L^–1^, which was then serially diluted. For the CCK-8 assay, cells treated with the extracts in 96-well plates were incubated for an additional 24 h. Then, each well was supplemented with 10 µL of CCK-8 reagent, incubated at 37 °C under 5% CO_2_ for 2 h, and the absorbance at 450 nm was measured using a microplate reader (EnSight, PerkinElmer, USA). For live-dead staining, cells in confocal dishes were first treated with extracts (15–30 mg L^–1^) for 24 h. Separately, Calcein AM (4 mM) and propidium iodide (PI, 16 mM) stock solutions were equilibrated at room temperature for 30 min. A working solution (2 µM Calcein AM, 8 µM PI) was then prepared by adding 5 µL of each stock to 10 mL of PBS. Finally, this working solution was applied to the cells for staining. Cells were washed with PBS, stained with working solution for 30–45 min at room temperature, then observed under confocal microscope (Leica TCS SP8, Leica Microsystems, Germany).

**S1.9 Cytotoxicity test**

To prepare a 2% erythrocyte suspension, 200 µL of anticoagulant was first placed in a microcentrifuge tube. Then, 2 mL of mouse whole blood was added and stirred with a glass rod for 10 min to remove fibrinogen, thus obtaining defibrinated blood. After adding 20 mL of PBS and mixing, the mixture was centrifuged at 1000–1500 rpm for 15 min, and the supernatant was discarded. The erythrocyte pellet was then washed 2–3 times with PBS under the same centrifugation conditions until the supernatant became colorless. Finally, a 2% (*v/v*) suspension was prepared by diluting 1 mL of the packed erythrocytes with 49 mL of PBS.

Eight EP tubes were numbered: 1–6 for test samples, 7 as negative control, 8 as positive control. The 2% erythrocyte suspension was mixed with PBS or deionized water and immediately incubated at (37 ± 0.5) °C. Hemolysis was monitored and recorded every 15 min in the initial phase, with photographs taken after 1 h. Subsequently, the supernatants were transferred to a 96-well plate, and the absorbance at 540 nm was measured using the microplate reader. The hemolysis rate was calculated as follows (S4):

$\text{Hemolysis rate (\%) }\text{=}\text{ }\text{[(}\text{As - An}\text{)/(}\text{Ap }\text{-}\text{ An}\text{)]×}\text{100\%}$ (S4)

where *A_h_*, *A_p_*, and *A_n_* denote the absorbance values of the sample (DCMP), the negative control (PBS), and the positive control (H_2_O), respectively.

**S1.10 In vitro coagulation test of the DCMP eutectogels**

Gauze and DCMP were formed into cylinders (8 mm in diameter, 5 mm in height) in 24-well plates. Before starting the experiment, all key components, including the calcium chloride solution, anticoagulated whole blood, and the 24-well plates, were preheated at 37 °C for 10 min to simulate physiological conditions. Immediately after sample preparation, 0.1 mL of recalcified whole blood (containing 10 mM CaCl_2_) was applied to the surface of each test sample, using gauze as the control. After incubation at 37 °C for 1, 2, 3, 4, and 5 min, respectively, 2 mL of deionized water was added to each well to lyse and remove unbound red blood cells. The absorbance of the resulting supernatant was then measured at 540 nm using a microplate reader. The absorbance of 100 μL recalcified whole blood diluted in 2 mL deionized water was measured and used as the reference standard corresponding to 100% hemolysis. The blood clotting index (BCI, %) was calculated using the following formula (S5):

$\text{BCI (\%) }\text{=}\text{ }\text{(}\text{OD}\text{2}\text{/}\text{OD}\text{1}\text{)×}\text{100\%}$ (S5)

where *OD_2_* is the absorbance value of the sample, and *OD_1_* is the absorbance value of the reference.

**S1.11 In vivo hemostatic assay of the DCMP eutectogels**

All animal procedures were approved by the Laboratory Animal Welfare and Ethics Committee of the Institute of health and Medicine, Hefei Comprehensive National Science Center (IHM-AP-2025-006-R5). The hemostatic properties of DCMP in vivo were evaluated by rat tail amputation hemorrhage model and rat liver perforation hemorrhage model. The study comprised four groups: a control group (no treatment), a gauze-treated group, a xenon lamp irradiation group, and a group treated with eutectogels combined with xenon lamp irradiation (wound temperature maintained at 60 °C during irradiation). There were 3 male Sprague-Dawley (SD) rats in each group (180–220 g).

*Tail amputation hemorrhage model in rats:* After anesthesia, SD rats were cut with scissors at a distance of 3 cm from the tail root of the rats. The wound hemostasis time and bleeding volume of the four groups of rats were recorded, i.e., the peeling filter paper was placed at the tail root to absorb blood, and then weighed as bleeding volume. Each rat was recorded and photographed separately.

*Liver perforation hemorrhage model in rats:* After anesthesia, a 2 cm incision was made in the chest after local shaving. SD rat liver was pulled out of the body, and a circular wound with a diameter of 8 mm was cut on the liver with a punch. The wound hemostasis time and bleeding volume of the four groups of rats were recorded (peeling filter paper was placed in the liver to absorb blood, and then weighed as bleeding volume). Each rat was recorded and photographed separately.

**S1.12 Ethical statement**

All animal procedures were approved by the Laboratory Animal Welfare and Ethics Committee of the Institute of health and Medicine, Hefei Comprehensive National Science Center (IHM-AP-2025-006-R5). Informed consent has been obtained from all participants or their legally authorized representatives for this study.

**S2 Results**


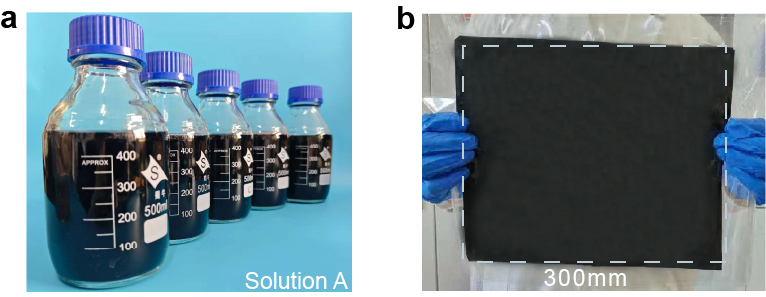


**Fig. S1** (**a**) Large-volume mixture solutions composed of DES, cellulose, and MXene. (**b**) Optical image of the large-area DCMP eutectogel


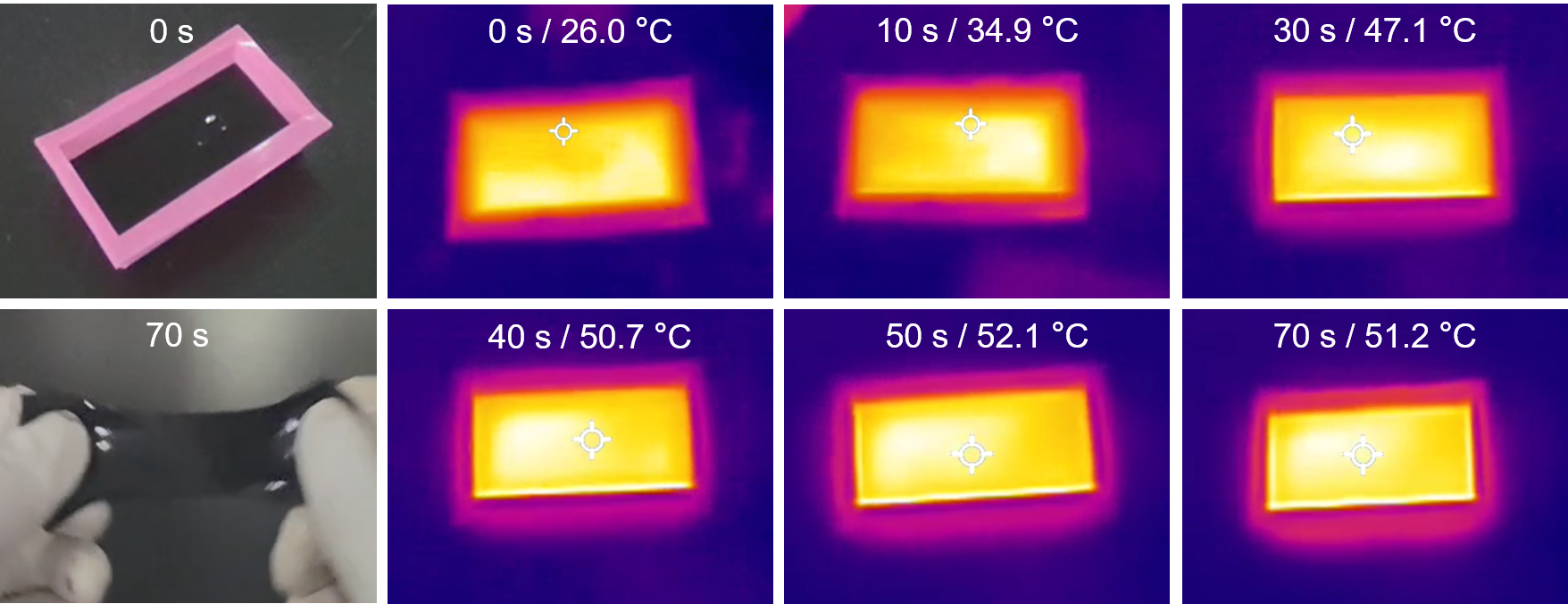


**Fig. S2** Optical and thermal imaging tracking the rapid formation of the DCMP eutectogels within 70 s


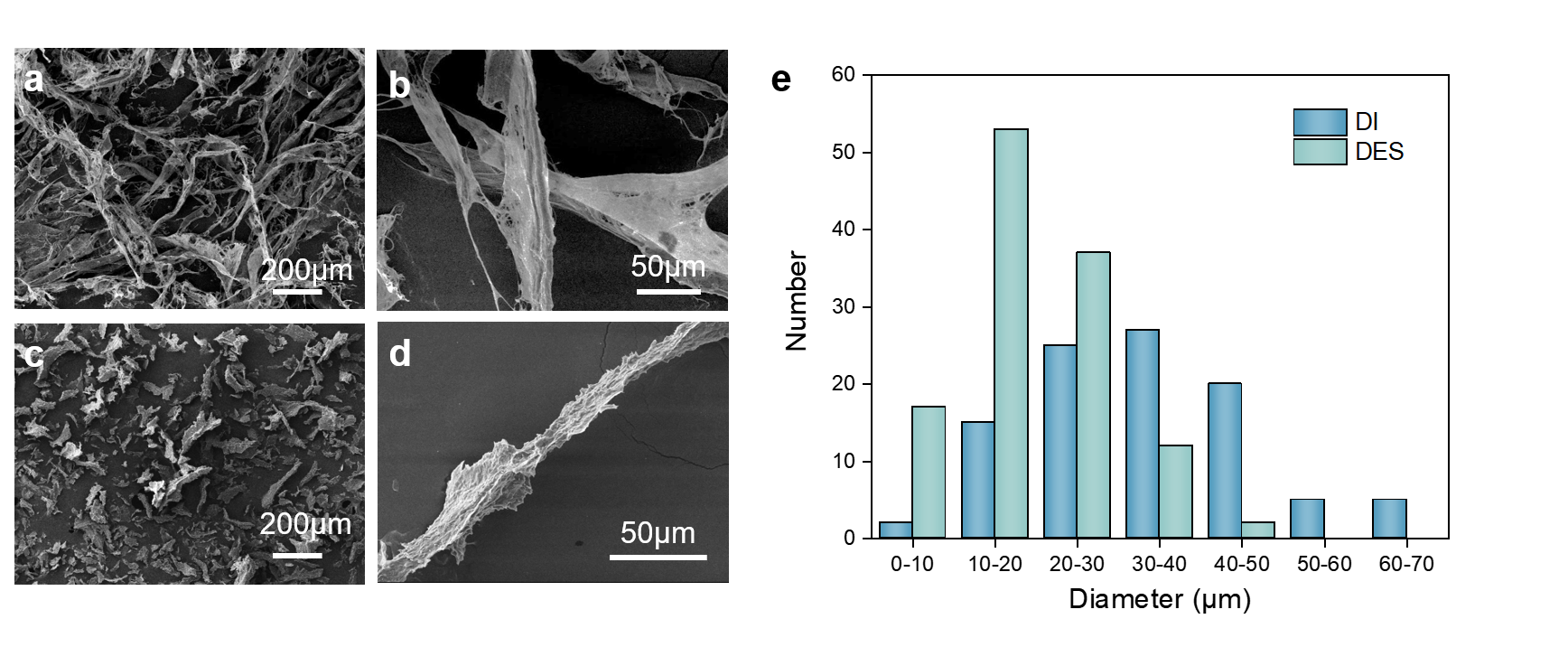


**Fig. S3** (**a, b**) SEM images of the cellulose fibers after ultrasonic treatment in deionized water (DI). (**c, d**) SEM images of the cellulose fibers after ultrasonic treatment in DES. (**e**) Diameter distribution of cellulose fibers after ultrasonic treatment in water and DES, respectively


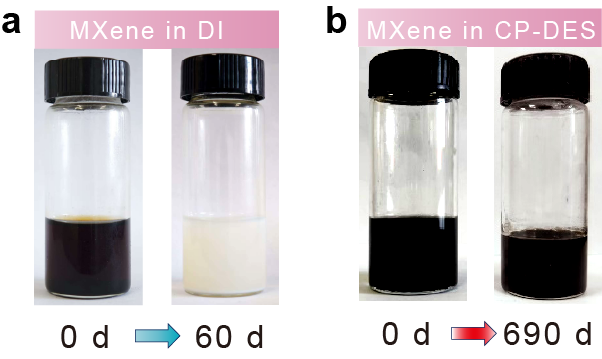


**Fig. S4** (**a**) Rapid oxidation of MXene in deionized water after 60 days. (**b**) Ultra-long-term stability of MXene dispersed in CP-DES after 690 days


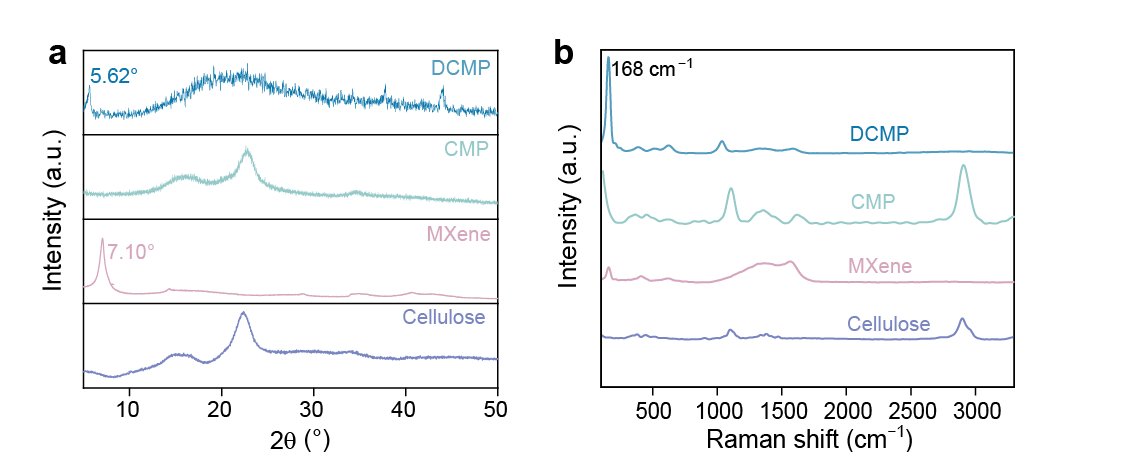


**Fig. S5** (**a**) XRD patterns of Cellulose, MXene, CMP, and DCMP. (**b**) Raman spectra of Cellulose, MXene, CMP, and DCMP


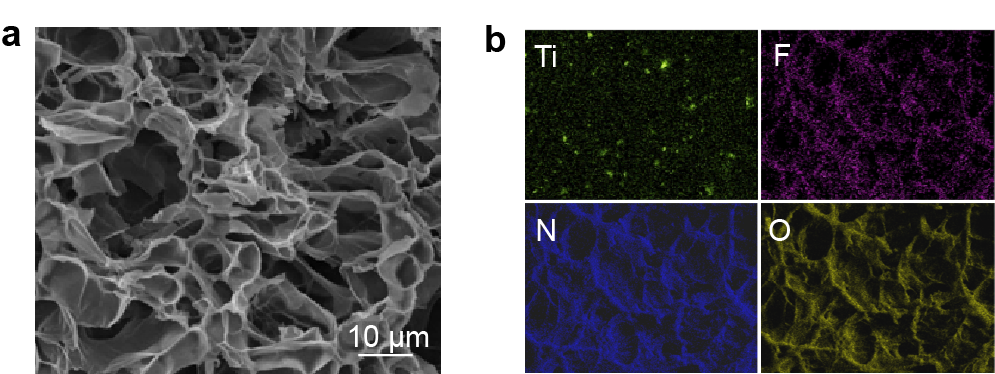


**Fig. S6 (a**) SEM image and (**b**) corresponding EDS elemental mapping (Ti, F, N, O) of the DCMP eutectogel

**Fig. S7** DSC curves of DCMP and CMP


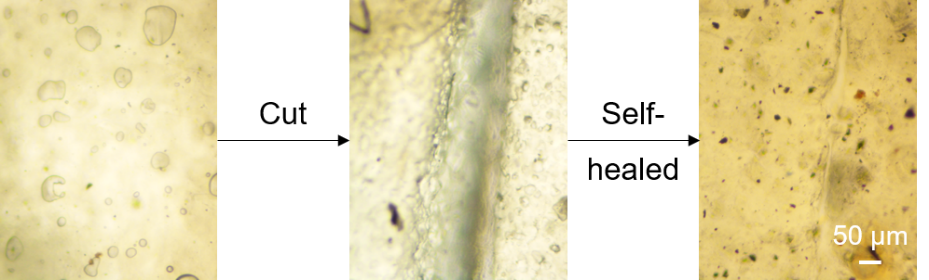


**Fig. S8** Optical microscopy images of the DCMP before and after self‑healing


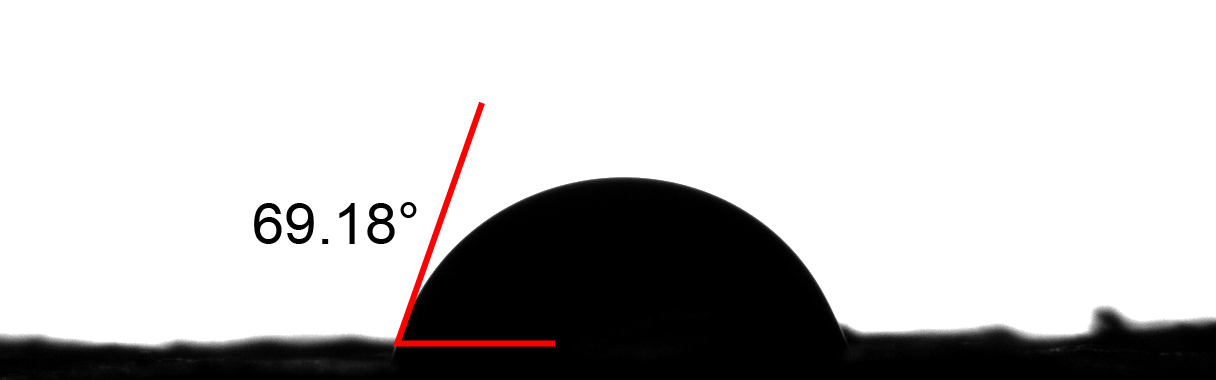


**Fig. S9** Static water contact angle measurement of the DCMP


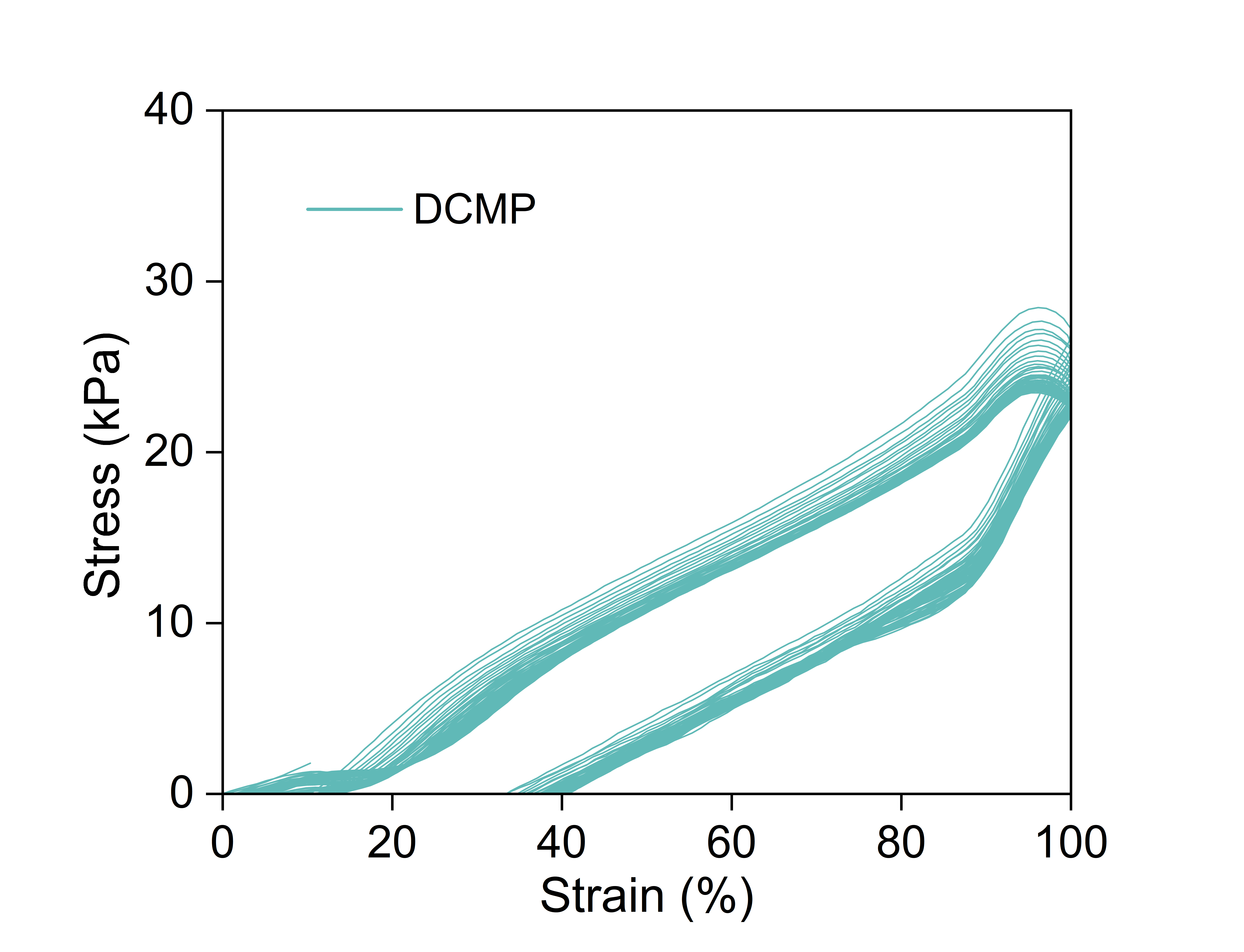


**Fig.** **S10** Stress-strain curves of DCMP eutectogel under 60 continuous loading-unloading cycles


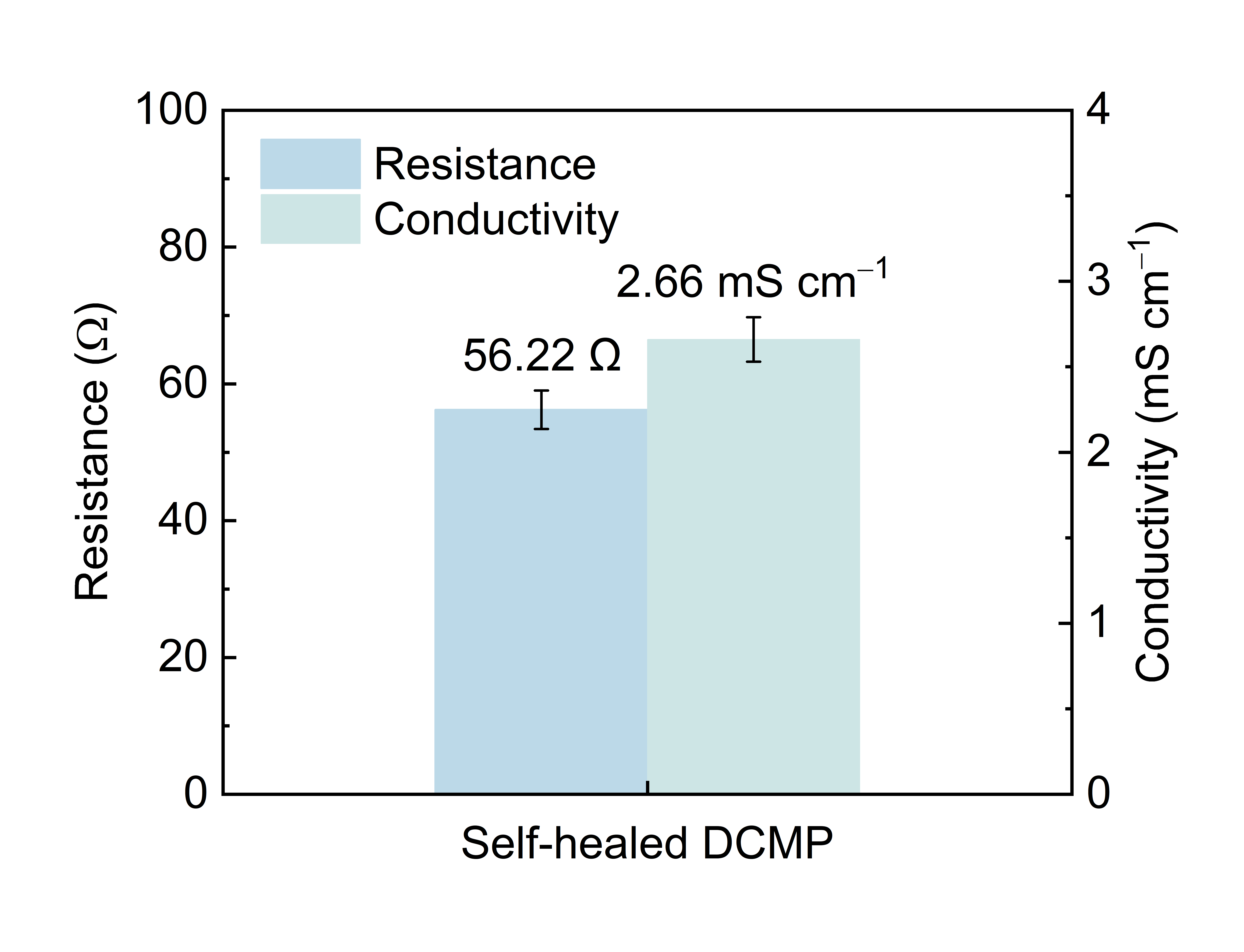


**Fig.** **S11** Internal resistance and conductivity of self-healed DCMP eutectogel


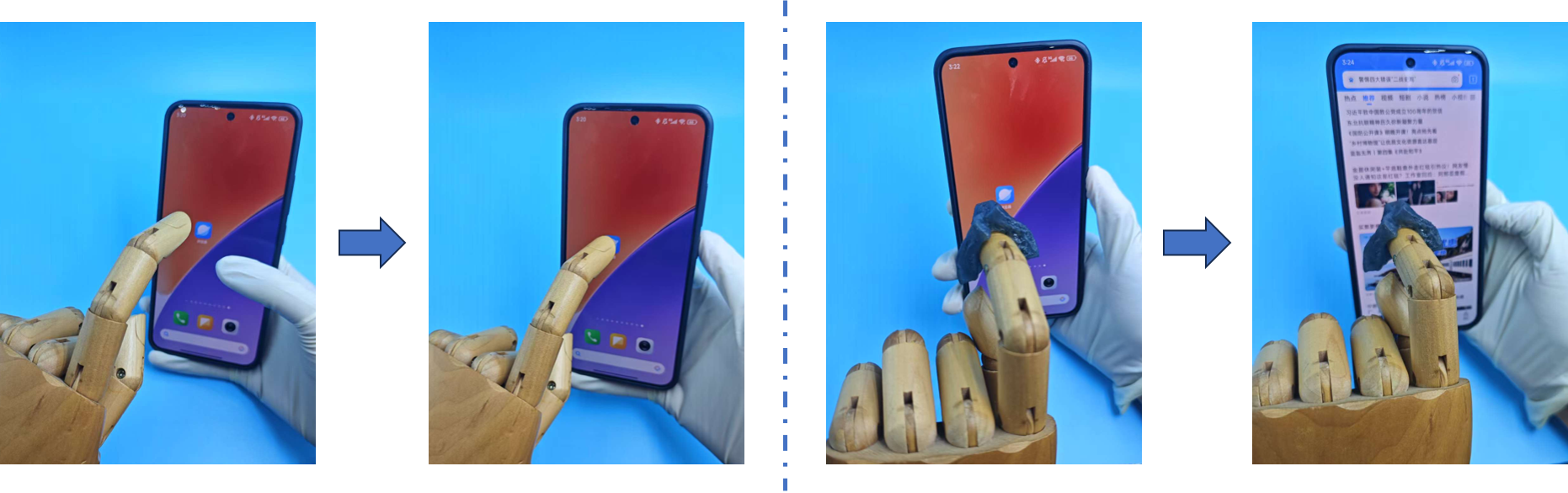


**Fig. S12** Demonstration of the ionic conductivity of the DCMP using a commercial smartphone.


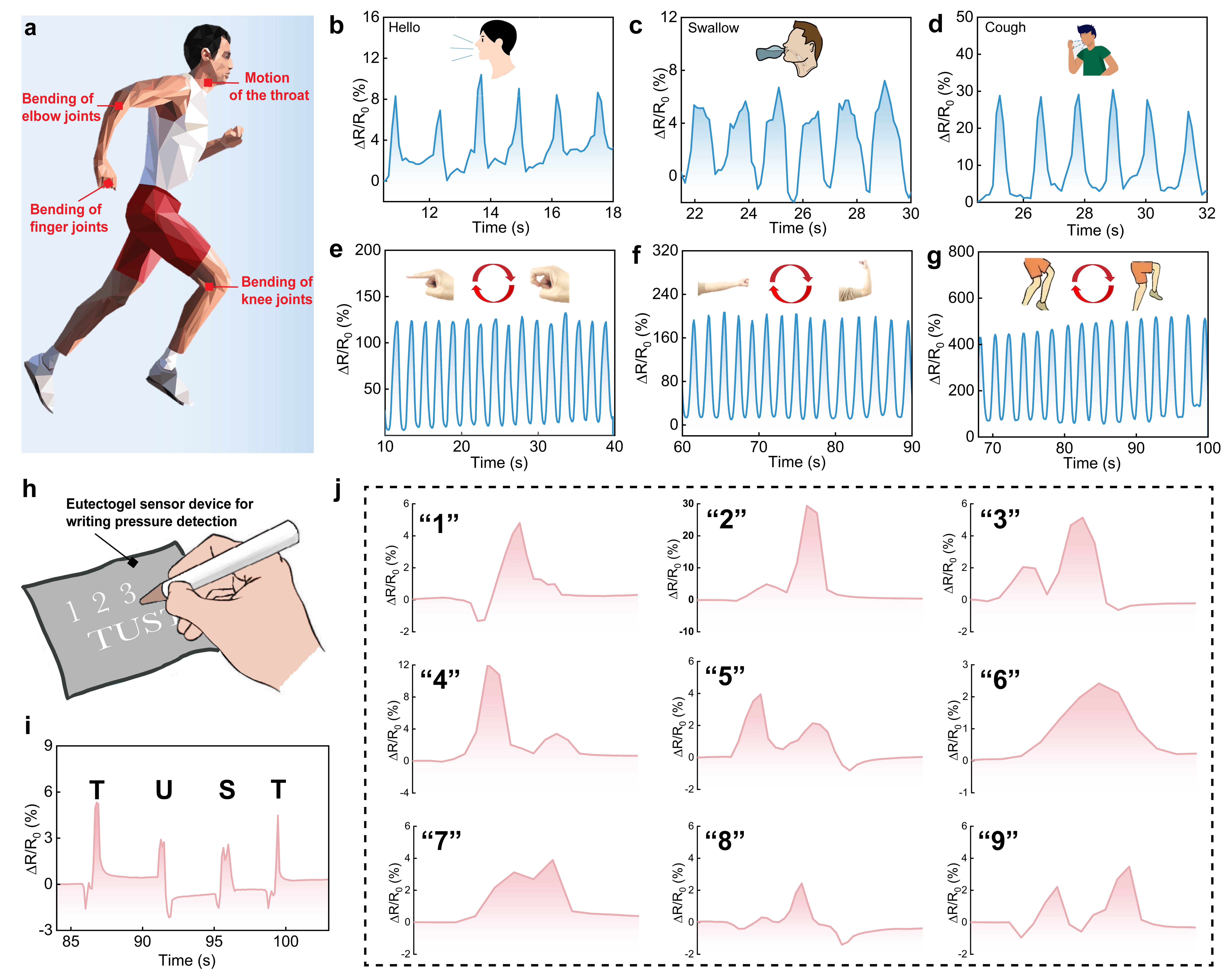


**Fig. S13** (**a**) Schematic diagram of the sensing performance test of DCMP in the detection of human motion. (**b**) Signal changes of saying "hello". (**c**) Signal changes of swallowing. (**d**) signal changes of coughing. (**e**) Signal changes of bent fingers. (**f**) Signal changes of elbow bending. (**g**) Signal changes of knee flexion. (**h**) The schematic diagram of the sensing performance test of DCMP in the detection of writing. (**i**) Response signals for writing "TUST". (**j**) Characteristic signal patterns for writing numbers "1–9"


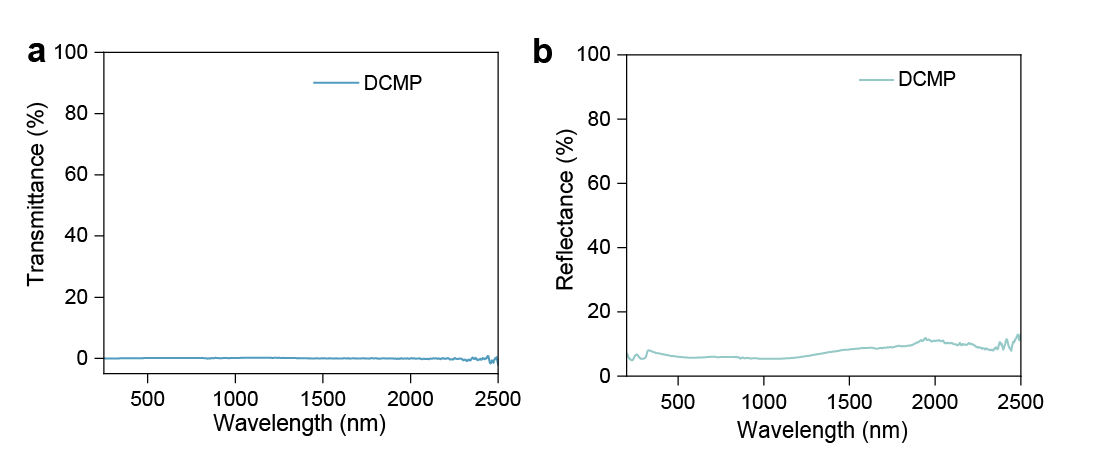


**Fig. S14** (**a**) Transmittance and (**b**) reflectance of DCMP


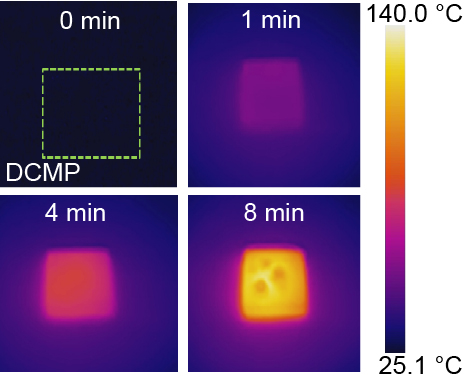


**Fig. S15** Thermal imaging image of DCMP under a radiation intensity of 0.3 W cm^–2^

**
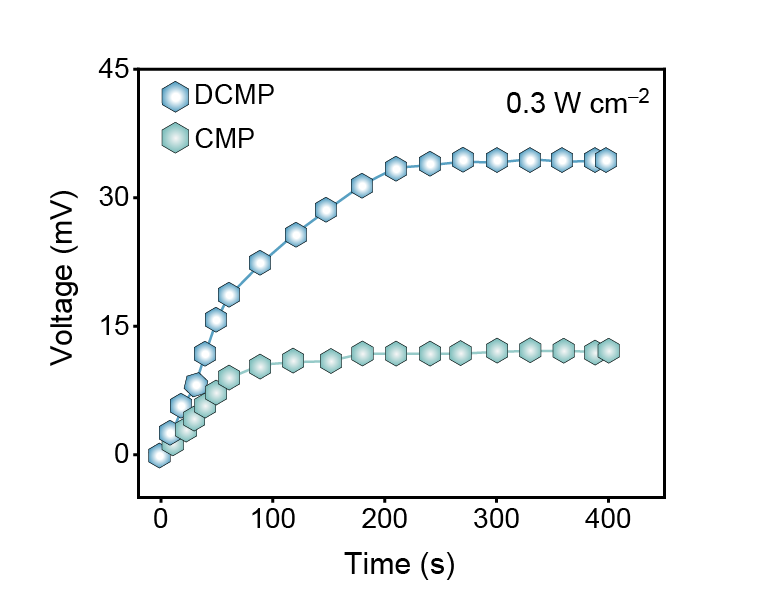
**

**Fig. S16** Time-dependent voltage changes of CMP and DCMP under a radiation intensity of 0.3 W cm^–2^

**Table S1** A comprehensive comparison between DCMP eutectogels and previously reported works in freezing resistance, conductivity, self-healing capability, gelation time and biocompatibility

| Sample | Conductivity (mS cm^–1^) | Freezing resistance (°C) | Biocompa-  tibility | Self-healing capability | 1/gelation time | Ref. |
| --- | --- | --- | --- | --- | --- | --- |
| DCMP | 2.99 | −50 | Yes | Yes | 70 s | This work |
| PETGs | 0.65 | −20 | Yes | Yes | 24 h | [S1] |
| MNOH | ≈0.18 | −40 | No | Yes | 2 d | [S2] |
| IHG | 2.40 | / | No | No | 30 min | [S3] |
| LSN-Fe/PAM | 1.60 | / | No | No | 60 s | [S4] |
| PSDIC-gel | 0.016 | / | No | Yes | 2 h | [S5] |

**Table S2** Comparison of mechanical properties of DCMP with previously reported eutectogels

| Sample | Tensile strain  (%) | Tensile stress  (kPa) | Compressive  strain (%) | Compressive  stress (kPa) | Ref. |
| --- | --- | --- | --- | --- | --- |
| DCMP | 757 | 217 | 70 | 115 | This work |
| ATH_6_ | ~880 | ~38 | 80 | ~111 | [S6] |
| PAA/DES/CNF | ~330 | 142 | 70 | ~150 | [S7] |
| PETGs | ~245 | 163 | - | - | [S8] |
| HPC-MDES | ~190 | ~140 | - | - | [S9] |

**Table S3** Comparison of photothermal conversion ability between DCMP eutectogels and previously reported works

| Sample | Intensity  (W cm^–2^) | Irradiation time (min) | Temperature  (℃) | Ref. |
| --- | --- | --- | --- | --- |
| DCMP | 0.1 | 15 | 45.7 | This work |
|  | 0.3 | 8 | 140 |  |
| eT-patch | 0.5 | 10 | 62.4 | [S10] |
| PM/C-EP-2 | 0.1 | 60 | 38.7 | [S11] |
| PPM@C-DES | 0.3 | 2 | 65 | [S12] |
| LAMC-C@M@P | 1.0 | 10 | 63.3 | [S13] |
| KMGHCa | 2.0 | 1.67 | 90 | [S14] |
| PG-Pg | 0.3 | 0.83 | 63 | [S15] |
| COL | 0.5 | 5 | 37.9 | [S16] |
| TFSFP | 0.1 | 10 | 40 | [S17] |
| PPN | 0.8 | 1.67 | 49 | [S18] |
| MPI-2 | 0.4 | 5 | 120 | [S19] |
| Cu-NP | 0.3 | 10 | 36 | [S20] |
| PNIPAM | 0.1 | 11 | 35 | [S21] |
| MXene/PAA | 0.3 | 5 | 42.5 | [S22] |
| PTC-HA | 2.0 | 15 | 69.4 | [S23] |
| CA-CT/CA | 0.1 | 15 | 40.2 | [S24] |
| FMMIHs | 0.1 | 5 | 35.0 | [S25] |
| MCGH | 0.1 | 30 | 38.6 | [S26] |
| Agar/PVA/MXene | 0.75 | 3.33 | ≈55 | [S27] |
| PNIPAm/CMC/CMCNT | 0.1 | 6 | 40.5 | [S28] |
| CZTS-PH | 0.1 | 10 | 34.8 | [S29] |
| PSS/PNIPAM | 1.01 | 0.33 | 60 | [S30] |
| CCHONCD | 0.3 | 3.5 | 36 | [S31] |
| NMPA-CS | 2.0 | 5 | 50 | [S32] |

**Table S4** Comparison of photoelectric conversion ability between DCMP eutectogels and previously reported works

| Sample | Intensity  (W cm^–2^) | Irradiation  time (s) | Electric current (μA) | Ref. |
| --- | --- | --- | --- | --- |
| DCMP | 0.1 | 20/450 | 11.5/37.3 | This work |
| PET | 0.1 | 20 | 22 | [S33] |
| STEIG | 0.1 | 250 | 2.54 | [S34] |
| G/ZnO@G/BN | 0.1 | 5 | 1.5 | [S35] |
| KM_15_G_8_H_0.5_Ca_5_ | 0.5 | 19 | 0.59 | [S14] |
| BPTP-5 | 0.5 | 20 | 0.57 | [S36] |
| Si-based photo-driven electronic skin | 0.5 | N/A | 0.016 | [S37] |

**Table S5** Hemostatic parameters (hemostatic time and total blood loss) of different groups in the rat tail-cutting model

| Group | No. | Hemostatic time (s) | Total blood loss (g) |
| --- | --- | --- | --- |
| Control | 1 | 167 | 0.496 |
|  | 2 | 176 | 0.533 |
|  | 3 | 188 | 0.666 |
| Gauze | 4 | 153 | 0.319 |
|  | 5 | 146 | 0.326 |
|  | 6 | 132 | 0.268 |
| Simulated sunlight | 7 | 142 | 0.329 |
|  | 8 | 133 | 0.266 |
|  | 9 | 135 | 0.257 |
| Eutectogels | 10 | 126 | 0.186 |
|  | 11 | 132 | 0.233 |
|  | 12 | 124 | 0.208 |
| Eutectogels+simulated sunlight | 13 | 98 | 0.134 |
|  | 14 | 102 | 0.149 |
|  | 15 | 114 | 0.166 |

Table S6 Hemostatic parameters (hemostatic time and total blood loss) of different groups in the liver perforation model

| Group | No. | Hemostatic time (s) | Total blood loss (g) |
| --- | --- | --- | --- |
| Control | 16 | 196 | 0.593 |
|  | 17 | 189 | 0.558 |
|  | 18 | 212 | 0.689 |
| Gauze | 19 | 129 | 0.456 |
|  | 20 | 116 | 0.426 |
|  | 21 | 109 | 0.331 |
| Simulated sunlight | 22 | 106 | 0.328 |
|  | 23 | 126 | 0.396 |
|  | 24 | 117 | 0.425 |
| Eutectogels | 25 | 98 | 0.267 |
|  | 26 | 102 | 0.301 |
|  | 27 | 106 | 0.315 |
| Eutectogels+simulated sunlight | 28 | 90 | 0.216 |
|  | 29 | 68 | 0.196 |
|  | 30 | 84 | 0.208 |

**Supplementary References**

1. Y. Shao, C. Dang, H. Qi, Z. Liu, H. Pei et al., Polyfunctional eutectogels with multiple hydrogen-bond-shielded amorphous networks for soft ionotronics. Matter **7**(11), 4076–4098 (2024). <https://doi.org/10.1016/j.matt.2024.09.009>
2. H. Liao, X. Guo, P. Wan, G. Yu, Conductive MXene nanocomposite organohydrogel for flexible, healable, low-temperature tolerant strain sensors. Adv. Funct. Mater. **29**(39), 1904507 (2019). <https://doi.org/10.1002/adfm.201904507>
3. W. Xiao, T. He, Q. Wang, X. Wang, Y. Lu et al., Low-hysteresis and tough ionohydrogels well-balanced by water. Adv. Funct. Mater. **35**(45), 2508300 (2025). <https://doi.org/10.1002/adfm.202508300>
4. H. Zhao, S. Hao, Q. Fu, X. Zhang, L. Meng et al., Ultrafast fabrication of lignin-encapsulated silica nanoparticles reinforced conductive hydrogels with high elasticity and self-adhesion for strain sensors. Chem. Mater. **34**(11), 5258–5272 (2022). <https://doi.org/10.1021/acs.chemmater.2c00934>
5. H. Ma, M. Wang, J. Hou, X. Wang, P. Sun et al., Strong and tough water-tolerant conductive eutectogels with phase-separated hydrophilic/hydrophobic dual ionic channels. Adv. Mater. **37**(14), 2500770 (2025). <https://doi.org/10.1002/adma.202500770>
6. T. Liu, Q. Wu, H. Liu, X. Zhao, X. Yi et al., A crosslinked eutectogel for ultrasensitive pressure and temperature monitoring from nostril airflow. Nat. Commun. **16**, 3334 (2025). <https://doi.org/10.1038/s41467-025-58631-7>
7. L.-H. Xu, Y.-T. He, Y. Xu, S. Sun, J. Liu et al., Deep eutectic solvent gel electrolytes reinforced with cellulose nanofibers for high-performance flexible solid-state supercapacitors. Adv. Funct. Mater. **35**(38), 2501263 (2025). <https://doi.org/10.1002/adfm.202501263>
8. Y. Shao, C. Dang, H. Qi, Z. Liu, H. Pei et al., Polyfunctional eutectogels with multiple hydrogen-bond-shielded amorphous networks for soft ionotronics. Matter **7**(11), 4076–4098 (2024). <https://doi.org/10.1016/j.matt.2024.09.009>
9. C. Lu, X. Wang, Y. Shen, S. Xu, C. Huang et al., Skin-like transparent, high resilience, low hysteresis, fatigue-resistant cellulose-based eutectogel for self-powered E-skin and human–machine interaction. Adv. Funct. Mater. **34**(13), 2311502 (2024). <https://doi.org/10.1002/adfm.202311502>
10. X. Ju, J. Kong, G. Qi, S. Hou, X. Diao et al., A wearable electrostimulation-augmented ionic-gel photothermal patch doped with MXene for skin tumor treatment. Nat. Commun. **15**, 762 (2024). <https://doi.org/10.1038/s41467-024-45070-z>
11. Z. Wei, Y. Wang, C. Cai, Y. Zhang, S. Guo et al., Dual-network liquid metal hydrogel with integrated solar-driven evaporation, multi-sensory applications, and electricity generation *via* enhanced light absorption and Bénard–Marangoni effect. Adv. Funct. Mater. **32**(41), 2206287 (2022). <https://doi.org/10.1002/adfm.202206287>
12. W. Wang, Z. Ma, Z. Hu, Y. Long, F. Wu et al., Synergistic enhancement of hole–bridge structure and molecular-crowding effect in multifunctional eutectic hydrogel strain/pressure sensor for personal rehabilitation training. Adv. Funct. Mater. **35**(39), 2502844 (2025). <https://doi.org/10.1002/adfm.202502844>
13. Y. Wang, K. Liu, W. Wei, H. Dai, A multifunctional hydrogel with photothermal antibacterial and AntiOxidant activity for smart monitoring and promotion of diabetic wound healing. Adv. Funct. Mater. **34**(38), 2402531 (2024). <https://doi.org/10.1002/adfm.202402531>
14. M. Hou, M. Yu, W. Liu, H. Zhang, Z. Wang et al., Mxene hybrid conductive hydrogels with mechanical flexibility, frost-resistance, photothermoelectric conversion characteristics and their multiple applications in sensing. Chem. Eng. J. **483**, 149299 (2024). <https://doi.org/10.1016/j.cej.2024.149299>
15. Y. Jiang, Y. Yang, X. Zheng, Y. Yi, X. Chen et al., Multifunctional load-bearing hybrid hydrogel with combined drug release and photothermal conversion functions. NPG Asia Mater. **12**, 18 (2020). <https://doi.org/10.1038/s41427-020-0199-6>
16. L. Zhang, G. He, Y. Yu, Y. Zhang, X. Li et al., Design of biocompatible chitosan/polyaniline/laponite hydrogel with photothermal conversion capability. Biomolecules **12**(8), 1089 (2022). <https://doi.org/10.3390/biom12081089>
17. C. Zhang, Y. Jiang, X. Zou, L. Xing, W. Liu et al., Biomass-based ferric tannate hydrogel with a photothermal conversion function for solar water evaporation. ACS Appl. Polym. Mater. **5**(11), 9574–9584 (2023). <https://doi.org/10.1021/acsapm.3c02022>
18. B. Lei, L. Cao, X. Qu, Y. Liu, J. Shao et al., Thermal-sensitive ionogel with NIR-light controlled adhesion for ultrasoft strain sensor. Nano Res. **16**(4), 5464–5472 (2023). <https://doi.org/10.1007/s12274-022-5151-3>
19. X. Wen, Z. Deng, H. Wang, J. Shi, S. Wang et al., High strength, self-healing sensitive ionogel sensor based on MXene/ionic liquid synergistic conductive network for human-motion detection. J. Mater. Chem. B **11**(47), 11251–11264 (2023). <https://doi.org/10.1039/D3TB01570J>
20. B. Tao, C. Lin, Y. Deng, Z. Yuan, X. Shen et al., Copper-nanoparticle-embedded hydrogel for killing bacteria and promoting wound healing with photothermal therapy. J. Mater. Chem. B **7**(15), 2534–2548 (2019). <https://doi.org/10.1039/C8TB03272F>
21. Z. Zhao, Z. Zhang, Z. Zhu, X. Zou, Y. Zhao et al., Photothermal responsive hydrogel for adsorbing heavy metal ions in aqueous solution. Colloids Surf. A Physicochem. Eng. Aspects **651**, 129425 (2022). <https://doi.org/10.1016/j.colsurfa.2022.129425>
22. Y. Bai, Y. Lu, S. Bi, W. Wang, F. Lin et al., Stretchable and photothermal MXene/PAA hydrogel in strain sensor for wearable human-machine interaction electronics. Adv. Mater. Technol. **8**(9), 2201767 (2023). <https://doi.org/10.1002/admt.202201767>
23. G. Liu, B. Li, J. Li, J. Dong, V.E. Baulin et al., Photothermal carbon dots chelated hydroxyapatite filler: high photothermal conversion efficiency and enhancing adhesion of hydrogel. ACS Appl. Mater. Interfaces **15**(48), 55335–55345 (2023). <https://doi.org/10.1021/acsami.3c11957>
24. X. Jing, L. Chen, Y. Li, H. Yin, J. Chen et al., Synergistic effect between 0D CQDs and 2D MXene to enhance the photothermal conversion of hydrogel evaporators for efficient solar water evaporation, photothermal sensing and electricity generation. Small **20**(50), 2405587 (2024). <https://doi.org/10.1002/smll.202405587>
25. J. Zhao, Y. Li, H. Zhu, G. Li, L. Kang et al., A smart MXene-copolymeric molecularly imprinted hydrogel with dual-response and photothermal conversion performance for specific recognition of *cis*-diol compounds. Nano Res. **15**(3), 2764–2772 (2022). <https://doi.org/10.1007/s12274-021-3991-x>
26. X. Chen, P. Zhang, X. Chen, C. Luo, M. Chao et al., Salt-resistant MXene-charge gradient hydrogel evaporator with boosted water transport for efficient photothermal desalination. Desalination **592**, 118155 (2024). <https://doi.org/10.1016/j.desal.2024.118155>
27. Y. Zhang, Z. Xu, Y. Yuan, C. Liu, M. Zhang et al., Flexible antiswelling photothermal-therapy MXene hydrogel-based epidermal sensor for intelligent human–machine interfacing. Adv. Funct. Mater. **33**(21), 2300299 (2023). <https://doi.org/10.1002/adfm.202300299>
28. J. Mei, Y. Jin, L. Bai, X. Shang, W. Zeng, A *Mimosa*-inspired photothermal-responsive multifunctional hydrogel for passive solar-driven efficient water purification. J. Mater. Chem. A **11**(47), 26063–26074 (2023). <https://doi.org/10.1039/D3TA05272A>
29. P. Liu, L. Xu, Z.-Y. Wang, Y. Huo, Y.-B. Hu et al., A salt-resistant and antibacterial Cu_2_ZnSnS4-based hydrogel for high efficient photothermal distillation in seawater desalination and sewage purification. ChemSusChem **16**(15), e202300611 (2023). <https://doi.org/10.1002/cssc.202300611>
30. S. Cao, X. Tong, K. Dai, Q. Xu, A super-stretchable and tough functionalized boron nitride/PEDOT: PSS/poly(*N*-isopropylacrylamide) hydrogel with self-healing, adhesion, conductive and photothermal activity. J. Mater. Chem. A **7**(14), 8204–8209 (2019). <https://doi.org/10.1039/C9TA00618D>
31. T. Chen, T. Yao, H. Peng, A.K. Whittaker, Y. Li et al., An injectable hydrogel for simultaneous photothermal therapy and photodynamic therapy with ultrahigh efficiency based on carbon dots and modified cellulose nanocrystals. Adv. Funct. Mater. **31**(45), 2106079 (2021). <https://doi.org/10.1002/adfm.202106079>
32. C.-W. Hsiao, H.-L. Chen, Z.-X. Liao, R. Sureshbabu, H.-C. Hsiao et al., Effective photothermal killing of pathogenic bacteria by using spatially tunable colloidal gels with nano-localized heating sources. Adv. Funct. Mater. **25**(5), 721–728 (2015). <https://doi.org/10.1002/adfm.201403478>
33. H. Yang, S. Ahmed Khan, N. Li, R. Fang, Z. Huang et al., Thermogalvanic gel patch for self-powered human motion recognition enabled by photo-thermal-electric conversion. Chem. Eng. J. **473**, 145247 (2023). <https://doi.org/10.1016/j.cej.2023.145247>
34. Y.-B. Xue, H.-T. Jiang, P. Luo, H.-J. Liu, Y.-H. Yang et al., Wearable solar ionic thermoelectric detectors for human motion monitoring and language recognition conversion. Adv. Funct. Mater. **35**(27), 2422592 (2025). <https://doi.org/10.1002/adfm.202422592>
35. P. Kang, F.I.T. Petrescu, Y. Wu, Y. Li, X. Li et al., A novel photo-thermal-electric conversion system with an integrated support material. Nanomaterials **13**(8), 1301 (2023). <https://doi.org/10.3390/nano13081301>
36. C. Sang, S. Wang, X. Jin, X. Cheng, H. Xiao et al., Nanocellulose-mediated conductive hydrogels with NIR photoresponse and fatigue resistance for multifunctional wearable sensors. Carbohydr. Polym. **333**, 121947 (2024). <https://doi.org/10.1016/j.carbpol.2024.121947>
37. J. Tian, J. Li, Z. Wu, L. Yin, X. Sheng et al., Photo-driven electronic skin enable activation of calcium channel for refractory wound healing. Nano Today **62**, 102697 (2025). <https://doi.org/10.1016/j.nantod.2025.102697>
